# Isolation and Characterization of *Enterococcus faecalis-*Infecting Bacteriophages From Different Cheese Types

**DOI:** 10.3389/fmicb.2020.592172

**Published:** 2021-01-08

**Authors:** Beatriz del Rio, Esther Sánchez-Llana, Noelia Martínez, María Fernández, Victor Ladero, Miguel A. Alvarez

**Affiliations:** ^1^Department of Technology and Biotechnology of Dairy Products, Dairy Research Institute, IPLA-CSIC, Villaviciosa, Spain; ^2^Molecular Microbiology Group, Instituto de Investigación Sanitaria del Principado de Asturias (ISPA), Oviedo, Spain

**Keywords:** bacteriophage, genome characterization, cheese, *Enterococcus faecalis*, lactic acid bacteria

## Abstract

Enterococci are a diverse group of Gram-positive, lactic acid bacteria (LAB). They are found in many environments, including fermented foods, in which they could constitute a health threat since they produce biogenic amines, which consumption can lead to intoxication. Moreover, enterococci has also emerged as an important hospital-acquired pathogens via its acquisition of antimicrobial resistance. Bacteriophages possess features that make them optimal biotechnological weapons for controlling bacterial growth in disease and food spoilage contexts. However, no silver bullet bacteriophage exists that can eliminate all the undesirable bacteria in a complex environment. Rather, a combination of phages with different host ranges would be required which implies the need for large collections of diverse phages. This work reports the isolation of several *Enterococcus faecalis*-infecting bacteriophages from different types of cheese, along with the range of *E. faecalis* strains of diverse origin (from dairy to clinical environments) they are able to infect. The isolated phages showed a large diversity regarding the number and origin of strains they infect. Some of these phages were subjected to morphological and genomic characterization which confirmed their diversity and showed they belong to different families and genera. The present findings increase the potential arsenal for the bacteriophage-based biocontrol of harmful *E. faecalis* populations.

## Introduction

The enterococci are a diverse group of Gram-positive belonging to the lactic acid bacteria (LAB) group. They are found in many environments, including the human gut where they normally exist as commensal microorganisms, although over the last 20 years they have emerged as important hospital-acquired pathogens through the acquisition of antimicrobial resistance (Torres et al., 2018). Nonetheless, they still play an important role in the production of fermented foods and some strains have even been proposed as probiotics (Foulquie Moreno et al., 2006). The presence of enterococci in different foods reflects their resistance to adverse environmental conditions: they can grow under aerobic and anaerobic conditions, over wide ranges of pH and temperature, and even in the presence of high salt concentrations – conditions commonly encountered in food production and storage (Foulquie Moreno et al., 2006). Moreover, they can even survive pasteurization (Ladero et al., 2011).

These bacteria, however, also improve the microbiological safety of dairy products. For example, many strains of enterococci, especially those of *Enterococcus faecium* and *Enterococcus faecalis*, produce bacteriocins against foodborne pathogens such as *Listeria monocytogenes* (Khan et al., 2010). They also contribute to their organoleptic characteristics, especially those of traditional Mediterranean cheeses (Ogier and Serror, 2008). A variety of proteolytic enzymes involved in casein degradation and amino acid metabolism, plus lipolytic and citrate-degrading enzymes found in enterococci strains contribute to the unique taste and flavor of each (Giraffa, 2003). Unfortunately, these bacteria may also produce undesirable biogenic amines (BAs) such as tyramine and putrescine (Ladero et al., 2010b, 2012a; Linares et al., 2011). Indeed, the production of these two BAs is a species-specific trait of *E. faecalis* (Ladero et al., 2012b). The consumption of foods with high content of these BAs can lead to headaches, migraines and hypertension (Ladero et al., 2010a; Wunderlichová et al., 2014). Recent studies have also shown tyramine may be genotoxic for intestinal cells (del Rio et al., 2018), and confirm tyramine and putrescine to have cytotoxic effects (Linares et al., 2016; del Rio et al., 2019a).

*E. faecalis* enters the cheese-making process either directly via the milk, as a contaminant of water, or via the cheese manufacturing and processing equipment (Giraffa, 2003). The capacity of these bacteria to resist the harsh conditions of the cheese-making environment, plus their ability to survive pasteurization, make it difficult to reduce their numbers without negatively affecting those of desirable LAB species useful in cheese production and ripening (Novella-Rodriguez et al., 2002; Ladero et al., 2011). Methods that specifically target *E. faecalis*, without affecting other LAB species present in the cheese matrix, are therefore needed.

In recent years, bacteriophages, i.e., viruses that specifically kill bacteria, have appeared as new biotechnological tools for reducing the population of specific foodborne pathogens and spoilage microorganisms (Garcia et al., 2008; Moye et al., 2018). They have even been studied as a means of reducing the population of BA-producing bacteria in foods, and consequently keeping the BA concentration at safer levels (Ladero et al., 2016a; Yamaki et al., 2018). Phages, as natural predators of the bacteria they infect, possess various characteristics that made them optimal biotechnological weapons to control bacterial population growth (Hesse and Adhya, 2019). They are harmless to humans, animals and plants, can resist the harsh environmental conditions of food processing, are relatively cheap and easy to isolate and propagate and, since they are self-replicating, only small doses are needed. Moreover, they are self-limiting; they can only multiply if the host is present (Sillankorva et al., 2012; Fernandez et al., 2017). However, there is no single type of phage that can infect and eliminate all the undesirable bacterial species found in cheese. This is certainly true if the target belongs to the LAB group. The phages that infect these species usually have very narrow host ranges, sometimes even just a single strain (Kot et al., 2014; Martínez et al., 2016). To be an effective technology, combinations of phages with different characteristics and different host ranges would need to be used.

Currently, few *E. faecalis* phages have been described and characterized at the genomic and functional level; many more must be investigated if they are to be used as a means of reducing BA in dairy products (Bolocan et al., 2019). The present work reports the isolation of *E. faecalis*-infecting bacteriophages from different cheeses, and the *E. faecalis* strains from dairy and clinical environments that they are able to infect. A sample of these phages based on their host ranges was selected for morphological and genomic characterization.

## Materials and Methods

### Material, Bacterial Strains, and Culture Conditions

Unless otherwise stated, all the reagents used in this work were purchased from Sigma-Aldrich (Spain). Sixty cheese samples belonging to nine cheese types, purchased at different markets over a 3-year period in Asturias (Spain), were used as a source of bacteriophages (Table 1). Different *E. faecalis* strains (15a, 19a, 23a, 52c, 63c, CECT795, V583 and CECT481^T^) of different origin were used as potential hosts in phage screening (Table 2). To determine the host range of isolated phages, additional *E. faecalis* strains were employed (Table 2). All bacterial strains were grown in M17 broth (Oxoid, Spain) supplemented with 0.5 glucose (GM17) without aeration. For phage enrichment cultures and host determination assays, the culture medium was supplemented with 10 mM Ca(NO_3_)_2_ and 10 mM Mg_2_SO_4_ (CM-GM17). Phage titers were determined in double-layer agar plates, mixing 100 μl of serial dilutions of the phage suspensions in SM buffer (20 mM Tris-HCl pH 7.5, 1 mM Mg_2_SO_4_, 100 mM NaCl) with 100 μl of an overnight culture of the target host strain. Plates were incubated at 30°C for 18 h and the resulting plaques counted.

**TABLE 1:** Phage screening results.

Cheese variety	Milk treatment	Number of samples screened	Isolated phages
Cabrales	Raw	16	141, 152, 153, 155, 158, 159, 160, 161
Emmental	Pasteurized	10	143, 146
Sheep milk cheese	Raw/Pasteurized	8	156, 157
Blue-veined	Pasteurized	8	
Boffard	Raw	7	142, 144, 145, 150, 151, 153
Gamoneu	Raw	4	140
Zamorano	Raw	3	147, 148, 149
Artisanal	Pasteurized	2	
Goat milk cheese	Raw/Pasteurized	2	Q69
Total		60	23

**TABLE 2:** *Enterococcus faecalis* strains used in the initial screening and to determine the isolated bacteriophages host ranges.

*E. faecalis* strains	Origin	Use	References/collection
CECT481^T^	Type strain	Screening	CECT
15a	Dairy	Screening	Ladero et al., 2012b
18a	Dairy	Host range	Ladero et al., 2012b
19a	Dairy	Screening	Ladero et al., 2012b
23a	Dairy	Screening	Ladero et al., 2012b
28a	Dairy	Host range	Ladero et al., 2012b
52c	Dairy	Screening	Ladero et al., 2012b
54c	Dairy	Host range	Ladero et al., 2012b
57c	Dairy	Host range	Ladero et al., 2012b
63c	Dairy	Screening	Ladero et al., 2012b
BA62	Dairy	Host range	Ladero et al., 2012b
BA64	Dairy	Host range	Ladero et al., 2012b
CECT 4039	Dairy	Host range	CECT
V61	Dairy	Host range	Ladero et al., 2012b
V63	Dairy	Host range	Ladero et al., 2012b
LMG20645	Meat	Host range	LMG
LMG12161	Meat	Host range	LMG
CECT795	Human	Host range	CECT
CECT4176	Human	Host range	CECT
HFS25	Human	Host range	Ladero et al., 2009
HFS57	Human	Host range	Ladero et al., 2009
HFS59	Human	Host range	Ladero et al., 2009
HFS62	Human	Host range	Ladero et al., 2009
HFS66	Human	Host range	Ladero et al., 2009
HFS69	Human	Host range	Ladero et al., 2009
JH2-2	Clinical	Host range	Giard et al., 1996
V583	Clinical	Screening	Paulsen et al., 2003

*CECT, *Colección Española de Cultivos Tipo*.LMG, *Laboratorium voor Microbiologie.**

### Isolation of *E. faecalis* Bacteriophages From Cheese

Phages were isolated from the cheeses by enrichment culture as previously described (Ladero et al., 2016a). Briefly, 1 g of each cheese sample was homogenized in 9 ml of 2% sodium citrate in a Lab-Blender 400 stomacher (Seward Ltd., United Kingdom). Hundred microliter of this homogenate were then added to 10 ml of CM-GM17 supplemented with cycloheximide (20 μg/ml) to inhibit yeast and mold growth, and inoculated into 100 ml of an overnight culture of single different host *E. faecalis* strains (Table 1). Enrichment cultures were incubated for 24 h. Samples were then centrifuged (2000 × *g* for 15 min) in a 5810 Eppendorf benchtop centrifuge, and 100 μl of the resulting supernatant used to inoculate (1% v/v) a new enrichment culture. After two rounds of enrichment, 10 μl were spotted onto double-layered agar CM-GM17 plates and incubated for 24 h at 30°C. If an inhibition halo was observed, the source supernatant was streaked to obtain single plaques. If differences in the morphology of the plaques, such as size or turbidity, were observed, host range comparisons were performed to see whether different phages were present. Phages from different host strain enrichment cultures, but from the same cheese sample, that showed an identical host range, were considered isolates of the same phage. However, if differences in the host range were observed, they were considered different phages. For bacteriophage purification, a single plaque was picked up with a sterile tip, inoculated into 50 ml of CM-GM17 broth containing the host strain, and incubated at 30°C until cell lysis was observed. The culture was then spun (10,000 × *g* for 15 min) in a 7780 centrifuge (Kubota, South Korea) with an AG6512C rotor, concentrated using the PEG/NaCl method (Binetti et al., 2005), and stored for further analysis.

### Microbiological Assays

The host range of the isolated phages was determined by challenging them against 27 *E. faecalis* strains of different origin (Table 2 and Supplementary Table 1). A 10 μl drop of phage suspension was placed on double-layered agar CM-GM17 plates containing the different potential host strains as previously described (del Rio et al., 2019b).

### Electron Microscopy

Concentrated particles of selected phages were purified in a continuous CsCl gradient by centrifugation (100,000 × *g* for 20 h at 4°C) in an Optimax ultracentrifuge (Beckman Coulter, United States), as described by Gutierrez et al. (2011). Purified phage particles were stained with 2% uranyl acetate solution and electron micrographs produced using a CCD Gatan Erlangshen ES 1000 W camera coupled to a JEOL JEM 1011 transmission electron microscope (JEDL USA Inc, United States) operated at 100 kV (performed at the Electron Microscopy Service of the Biotechnology National Centre [CNB-CSIC], Spain).

### Phage DNA Extraction

Phage DNA was obtained from a concentrated suspension of phage particles as previously described (del Rio et al., 2019b). Briefly, 80 μl of lysis solution (0.25 M EDTA, pH 8.1; 0.5 M Tris-HCl, pH 9.6; 2.5% sodium dodecyl sulfate) were added to 400 μl of phage suspension and incubated in a water bath at 65°C for 30 min. Hundred microliter of 8 M potassium acetate were then added, and the mixture incubated on ice for 15 min and further centrifuged (16,000 × *g*, 10 min, at 4°C) in a 5415R Eppendorf centrifuge. Phage DNA was precipitated from the supernatant with one volume of isopropanol, kept at room temperature for 5 min, and centrifuged again (16,000 × *g*, 10 min at room temperature). The pellet was resuspended in TE buffer (10 mM Tris-HCl, 1 mM EDTA, pH 8.0) in the presence of 0.3 M sodium acetate pH 4.8, and precipitated twice with isopropanol for 5 min, followed by centrifugation (16,000 × *g*, 10 min at room temperature). The DNA pellet was then washed with absolute ethanol and 70% ethanol before being resuspended in TE buffer.

### Whole Genome Sequencing and Annotation

A genomic library of 0.5 kbp was constructed and subjected to 150 paired-end sequencing (providing approximately 800-fold coverage) using a HiSeq 1000 System sequencer (Illumina) at GATC Biotech (Germany). Quality filtered reads were assembled using SPADES software^1^ (Bankevich et al., 2012). For vB_EfaS_159, a second library was constructed using fragments generated after *Hin*dIII digestion of the genomic DNA. Both libraries were combined in the assembly using SPADES software. The genomic sequence was closed by Sanger sequencing (performed at Macrogen, Spain) after PCR amplification following previously described procedures (Perez et al., 2015). Annotation was performed using the RAST server^2^ (Aziz et al., 2008), improving it with results obtained from BLAST analysis^3^ (Altschul et al., 1997). The genome sequences obtained were deposited at the European Nucleotide Archive (ENA) under accession numbers CAJCJZ010000002.1 (140), CAJDJZ010000002.1 (149), CAJDKF010000002.1 (159), and CAJDJX010000002.1 (Q69).

### Phylogenetic Analysis

A phylogenetic analysis of complete *E. faecalis* bacteriophage genome sequences available in the NCBI database (Supplementary Table 2) was performed using the Neighbor joining method following genome nucleotide sequence alignment undertaken employing MAFFT v.7 software^4^ (Katoh et al., 2019). All genomic sequences were edited to start at the terminase, or at the major capsid genes if no terminase gene was identified. A second phylogenetic analysis was performed aligning the amino acidic sequence of the major capsid protein of the same phages by the unweighted pair group method with arithmetic means (UPGMA) using MAFFT v.7 software (Katoh et al., 2019). The trees were visualized in iTOL^5^ (Letunic and Bork, 2016).

## Results

### Bacteriophage Screening

A total of 60 retail cheese samples from different geographical origin purchased at different markets in Asturias (Spain) and with different technological characteristics were screened for the presence of *E. faecalis* infecting phages. After two rounds of enrichment culture, a drop of the supernatant was deposited on CM-GM17 agar plates and covered, individually, with the host strains used in enrichment. A growth inhibition halo was deemed to indicate the potential presence of a phage. Likely phage-positive supernatants were then streaked onto the plates, covered with the same inhibited strains, following the double-layered agar CM-GM17 method to check for the appearance of isolated lysis plaques. A single plaque was used to propagate and store phages for further characterization. Twenty-two *E. faecalis*-infecting phages were isolated from 15 cheese samples, i.e., 25% of the samples screened. In at least five samples, more than one phage was isolated (data not shown). The cheeses returning the most phage-positive results were Zamorano, Boffard, and Cabrales, the latter two accounting for 60% of all those isolated. Two of the isolated phages -156 and Q69- have been previously described (Ladero et al., 2016a; del Rio et al., 2019b) and have been included in this work for further characterization and comparison.

### Bacteriophage Host Range

To determine and compare the host ranges of the isolated phages, the battery of *E. faecalis* strains tested was increased to 27 to include those of meat and human origin (Table 2). All were challenged with the 22 phages using the spot test. If a clear halo was observed, the strain was considered sensitive to that phage. Phages 140, 148, 149, and 157 showed the widest host ranges, infecting more than 50% of the strains tested. Among the other phages, eight infected between 25 and 50% of the strains, and eight infected < 25% (Table 3 and Supplementary Table 1). It is noteworthy that phage 157 infected 75% of the strains challenged, while phages 142 and 155 infected just one strain. *E. faecalis* type strain CECT481^T^ was among the most sensitive of strains, together with two strains of human origin (infected by >70% of the tested phages). Although the screened samples were from dairy environments, only seven phages were able to infect > 50% of the tested *E. faecalis* dairy strains. Only two phages were able to infect > 50% of the challenged strains of human origin. None of the phages were able to infect the two strains of meat origin, while at least five phages infected the two strains of clinical origin (Table 3).

**TABLE 3:** Host range of the isolated phages.

		*E. faecalis* strain origin					
Phage	Plaque size	Type (*n* = 1)	Dairy (*n* = 14)	Meat (*n* = 2)	Human (*n* = 8)	Clinical (*n* = 2)	Total (*n* = 27)
140	++	1	11	1	5	1	19
141	++	1	6	0	2	0	9
142	+	0	0	0	1	0	1
143	+	0	1	0	3	2	6
144	++	0	2	0	4	1	7
145	++	0	2	0	3	1	6
146	++	0	1	0	1	2	4
147	++	0	1	0	3	2	6
148	+	1	12	1	2	1	17
149	+	1	12	1	2	1	17
150	++	1	6	0	2	1	10
151	++	1	2	0	0	1	4
152	++	1	4	0	0	0	5
153	+++	1	4	0	2	0	7
155	++	1	0	0	0	0	1
156	+	1	13	1	3	2	20
157	+	1	13	1	3	2	20
158	+++	1	7	0	2	0	10
159	+++	1	6	0	2	0	9
160	+++	1	7	0	2	0	10
161	+++	1	6	0	0	0	7
Q69	++	1	6	0	3	0	10

*The number of strains infected within each origin category (type, dairy, meat, human, and clinical) is indicated. +: tiny plaques; ++: small; +++: plaques > 1 mm diameter.*

### Bacteriophage Morphology

In addition to the previously characterized phages -156 and Q69-, we have selected three additional phages -140, 149, and 159- showing functional or differential structural traits for more in-depth characterization. Phage 140 was selected to further investigate the underlying genetic causes that make its genome resistant to cleavage by several restriction enzymes. Regarding phages 149 and 159, they were selected since the former had one of the largest host ranges (Table 3 and Supplementary Table 1) and the latter, the larger plaque size among the phages analyzed in this work (data not shown).

Morphology of phage Q69 has been reported previously (Ladero et al., 2016a), showing it to belong to the family *Siphoviridae*. Phage 156 was previously classified as a member of the *Myoviridae* family (del Rio et al., 2019b), but it has been reclassified as a member of *Herelleviridae*, genus Kochikohdavirus (Barylski et al., 2020).

**FIGURE 1:**
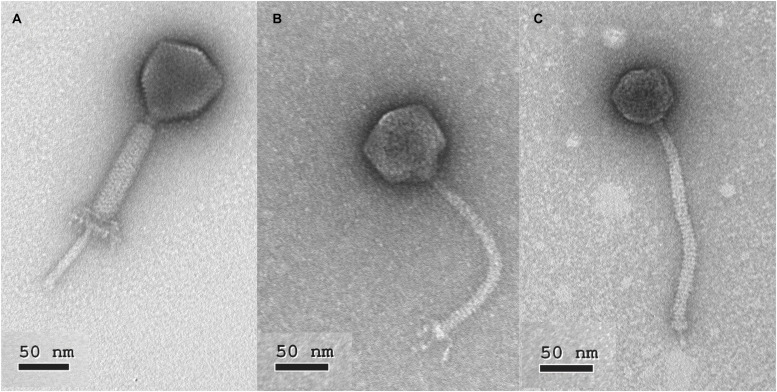
Electron photomicrographs of selected phages. **(A)** vB_EfaH149, **(B)** vB_EfaS_140, **(C)** vB_EfaS_159.

The morphology of phage 149 showed the characteristics typical of myoviruses, i.e., an icosahedral head with a non-flexible contractile tail (Figure 1A). The head diameter was estimated at 75 ± 3 nm, the contracted tail sheath at 114 ± 5 nm, and the visible section of the tail tube at 69 ± 4 nm, indicating an estimated tail length of 184 ± 6 nm. A neck and a baseplate were also clearly observed (Figure 1A). Based on phage 149’s genome (see next section) and its strong similarity with phage 156 and other genomes of *Kochikohdavirus* members, it is proposed here to be a member of the family *Herelleviridae*. Its proposed name following the International Committee on Taxonomy of Viruses (ICTV) recommendations is vB_EfaH_149.

The observed morphology of phage 140 showed it to belong to this same family: head diameter 71 ± 3 nm, flexible tail 172 ± 5 nm long, and a base plate with some accessory decorating proteins (Figure 1B). Based on these characteristics and following the ICTV recommendations it is here proposed that it be named vB_EfaS_140.

The morphology of phage 159 (Figure 1C) (tail length 190 ± 3 nm, the head diameter 57 ± 4 nm, baseplate and a clearly observable spike) indicates it to also belong to the family *Siphoviridae*. It is here proposed it be named vB_EfaS_159 following the ICTV recommendations.

### Bacteriophage Genomes

The genomes of the selected phages 140 (vB_EfaS_140), 149 (vB_EfaH_149), and 159 (vB_EfaS_159), plus that of Q69, were sequenced and characterized. Their G + C content ranged from 30.2% for phage vB_EfaS_140 to 36% for phage vB_EfaH_149, both below the G + C content of the *E. faecalis* host species (estimated at 37.4%).

**FIGURE 2:**
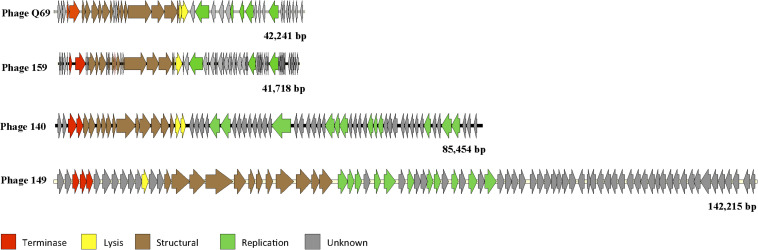
Diagram of the selected phage genomes. Each arrow represents an *orf*. The color code indicates the putative function assigned to each detected gene. The genome size (bp) is indicated.

The genome of phage vB_EfaH_149 was shown to be a dsDNA molecule of 142,215 bp with 193 open reading frames and six *tRNA* genes coding for sequences carrying the codons TGT, CAT, TGG, TCT, CCA and GTC (for threonine, methionine, proline, arginine, tryptophan, and aspartate respectively). Only 18% of the open reading frames coded for an identifiable function, all related to replication, packaging, capsid formation and lysis (Figure 2 and Supplementary Table 3a). The genome showed strong similarity (data not shown) to that of the previously sequenced phage 156, which consists of a 141,133 bp-long dsDNA molecule comprising 209 open reading frames and 5 *tRNA* genes (del Rio et al., 2019b).

Although phage Q69 has been previously described (Ladero et al., 2016a), its genome was not characterized and thus it was sequenced in this work. Q69 genome consist in a dsDNA molecule 42,241 bp long with 71 open reading frames. For 43% of these, a putative function was assigned – the largest proportion of functional assignments for all five genomes analyzed. The putative functions assigned were related to head, neck, and tail structural proteins, and replicative functions. In addition, a putative holin- and lysin- coding genes were found (Figure 2 and Supplementary Table 3b). The arrangement of the genes in the genome map revealed two divergently transcribed regions organized as functional modules (Figure 2).

The genome of phage vB_EfaS_140 was found to be a dsDNA molecule of 85,454 pb comprising 129 open reading frames and a *tRNA* gene coding for the CCA codon (the codon for tryptophan). A putative function could be assigned to only 25% of the open reading frames found, with functions related to replication, packaging, capsid formation and lysis (Figure 2 and Supplementary Table 3c). Among those genes for which a putative function was determined, some coded for enzymes related to nucleotide modification, such as CDS_73 coding for a putative DNA beta-glucosyltransferase similar to that of *Escherichia coli* phage T4 (Blastp *E* value 2e-75), which might be related to the observed refraction of its genomic DNA to enzymatic restriction with several tested nucleases (data not shown).

Finally, the genome of phage vB_EfaS_159 was also found to be a dsDNA molecule, 41,718 bp long, with 69 open reading frames. Putative functions involving replication, packaging, capsid formation and lysis functions were assigned to 34% of them (Figure 2 and Supplementary Table 3d).

No gene in any of the analyzed genomes was identified as toxin-encoding. Nor was any related to antibiotic resistance or any other pathogenic trait.

### Phylogenetic Relatedness of Bacteriophages

**FIGURE 3:**
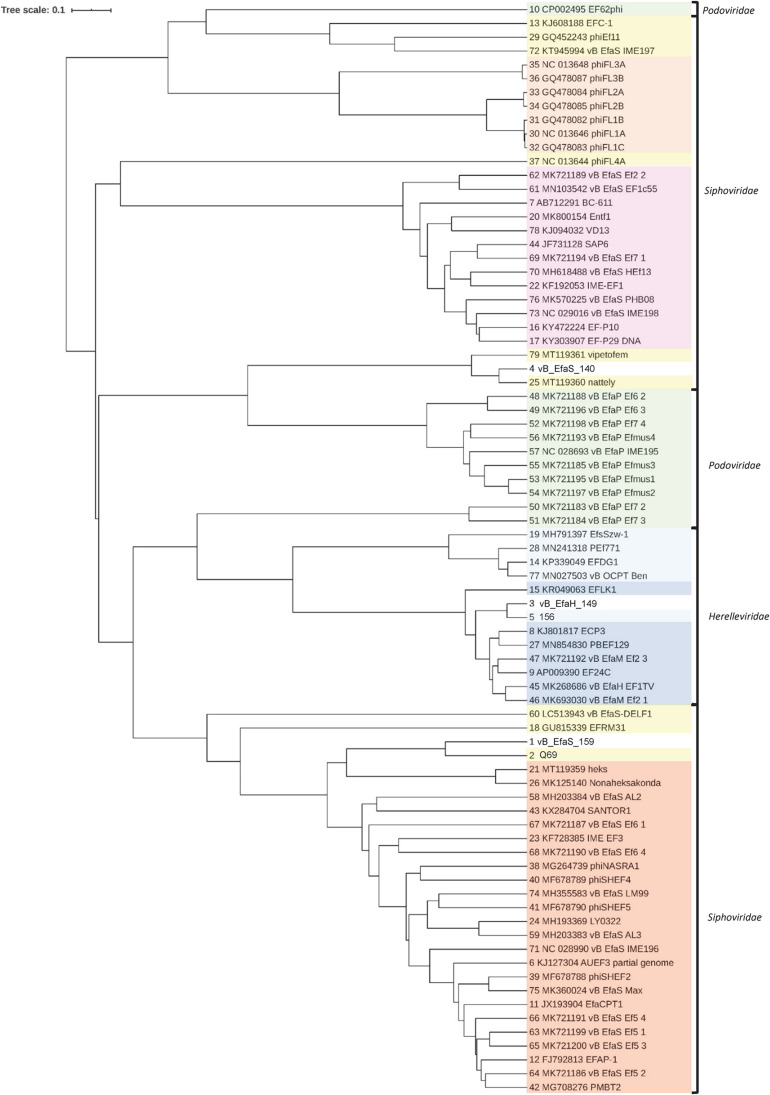
Phylogenetic tree based on the nucleotide sequences of the complete genomes of the selected phages and of *E. faecalis* phages with complete genomes in the NCBI database. The name of each phage is shown (as represented in Supplementary Table 2). 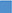 *Herelleviridae*, *Kochikohdavirus*; 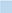 *Herelleviridae*, unclassified; 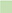 *Podoviridae*, *Picovirinae*; 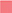 *Siphoviridae*, *Efquatrovirus*; 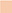 *Siphoviridae*, *Phifelvirus*; 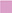 *Siphoviridae*, *Saphexavirus*; 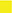 *Siphoviridae*, unclassified.

To study the diversity of the four sequenced genomes, a phylogenetic tree was constructed based on their alignment with the full genomes of *E. faecalis*-infecting phages available in the NCBI database (Figure 3 and Supplementary Table 2); this included the previously published 156 genome. The diversity of the database-available genomes was wide. Most of them grouped according to their taxonomic classification, although several discrepancies can be observed (Figure 3). The vB_EfaS_140 genome did not group with any of the other phage genomes from the present work, but did so with other *E. faecalis*-infecting phage genomes, all of which share the characteristic of being among the largest genomes of the *Siphoviridae* group (>85 kbp). The genomes of phages vB_EfaH_149 and 156 were closely related, as were those of Q69 and vB_EfaS_159.

**FIGURE 4:**
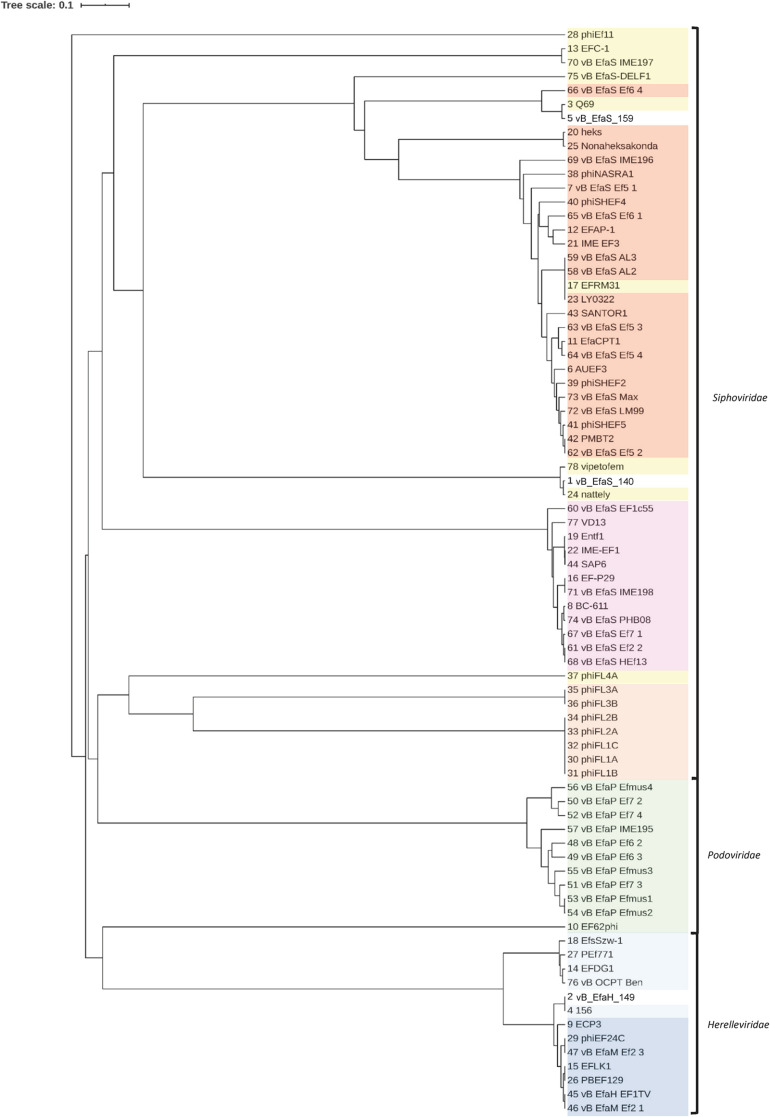
Phylogenetic tree based on the amino acid sequence of the major head proteins of the selected phages and of *E. faecalis* phages with complete genomes in the NCBI database. The name of each phage is shown (as represented in Supplementary Table 2). 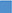 *Herelleviridae*, *Kochikohdavirus*; 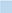 *Herelleviridae*, unclassified; 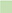 *Podoviridae*, *Picovirinae*; 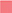 *Siphoviridae*, *Efquatrovirus*; 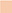 *Siphoviridae*, *Phifelvirus*; 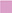 *Siphoviridae*, *Saphexavirus*; 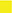 *Siphoviridae*, unclassified.

In further analysis, the major capsid proteins of the three selected phages plus 156 and Q69 were aligned with those of the *E. faecalis*-infecting phages available in the NCBI database (Figure 4 and Supplementary Table 2). The phylogenetic tree generated reflects the described taxonomy of the *E. faecalis*-infecting phages, which groups them into three families -*Herelleviridae*, *Podoviridae* and *Siphoviridae-* but also into subfamilies and genera (Supplementary Table 2). Some phages annotated as unclassified in the NCBI database (Supplementary Table 2) clustered with well described groups, indicating them to share common characteristics (Figure 4). Q69, shown in the NCBI database to be an unclassified *Siphoviridae* phage, grouped with phages belonging to the genus *Efquatrovirus*. Phage vB_EfaS_159, which is close to Q69, was also associated with members of *Efquatrovirus*. Phage vB_EfaS_140 grouped with the phages vipetofem and nattely, both unclassified *Siphoviridae* phages. All three share the characteristic of having the largest genomes among the *E. faecalis*–infecting *Siphoviridae* described (>80 kbp long). Phage vB_EfaH_149 grouped with family *Herelleviridae* genus *Kochikohdavirus* phages. The *Kochikohdavirus* genus comprises a group of phages, the genomes of which, show strong similarity. A high degree of homology was observed between the vB_EfaH_149 and 156 genomes indicating vB_EfaH_149 to be a new member of this genus.

## Discussion

Biogenic amine are toxic compounds that can accumulate by the microbial action at elevated concentrations in foodstuffs, especially in fermented food products such as cheese. In the particular case of cheese -one of the foods in which higher concentrations of BA can be found (EFSA, 2011)- the microorganisms responsible of BA accumulation are members of the LAB group (Ladero et al., 2016b), which play an essential role in the fermentation and determination of the organoleptic characteristics of the mature cheese (Beresford and Williams, 2004). This dual role implies that specific measures targeting BA-producing microorganisms without affecting the other LAB present in the cheese matrix must be adopted to ensure the quality and final organoleptic characteristics, but maintaining food safety for consumers. Among them, phage biocontrol has arisen as a promising biotechnological alternative (Garcia et al., 2008; Ladero et al., 2016a; Yamaki et al., 2018). In fact, two of the bacteriophages compared in this work, 156 and Q69, were successfully assayed in an experimental cheese model for the reduction of the final content in tyramine and putrescine -the BA most frequently found in cheeses- which are mainly produced by *E. faecalis* (Fernández et al., 2007; Ladero et al., 2016a; del Rio et al., 2019b). One of the problems that phage biocontrol must face to be applied to LAB is the narrow host range of the phages that infect bacteria of this group (Kot et al., 2014; Martínez et al., 2016). To overcome this problem, one of the solutions is to use phages cocktails instead of single phages (Chan et al., 2013; Bolocan et al., 2019). A requisite to design adequate phage cocktails targeting the large diversity of strains that can be found in a rich non-sterile matrix as milk, is to have a large collection of well characterized phages with different host ranges.

The cheeses made from raw milk provided the largest number of different phages (Table 1) (but note that some types of cheese were more extensively sampled than others). This is to be expected since cheeses made from raw milk have a greater diversity of microorganisms, especially of secondary microbiota such as enterococci, including *E. faecalis* (Gobbetti et al., 2018). Only five phages were able to infect more than half of the strains challenged (Table 3). Among them, phage vB_EfaH_149 showed typical myovirus morphology, and belongs to the new *Herelleviridae* family, the phages of which usually show wider host ranges than *Siphoviridae* phages. No clear relationship was seen between the origin of the phages and the origin of the *E. faecalis* strains they infected. It might be expected that the dairy phages vB_EfaH_149 and 156 would preferentially infect *E. faecalis* strains of dairy origin (Table 3). The more *E. faecalis* strains a particular phage can infect, the greater the diversity of host origin. Interestingly, some of the isolated phages were able to infect clinical *E. faecalis* strains, including AMR strains (Table 3). Thus, they may be worth investigating as phage therapy agents. A large proportion of the isolated phages were unable to infect half of the strains tested, regardless of their origin. However, this is not surprising since the phages already known to infect LAB species are reported to have a narrow host range (Kot et al., 2014; Martínez et al., 2016). Since several of these belong to different families (or even genera) and have different host ranges, the selection of an adequate combination would improve the range of *E. faecalis* strains against which a cocktail could be effective (Chan et al., 2013).

Phage 156 was previously classified as a member of *Myoviridae* (del Rio et al., 2019b), but the ICTV recently published a new family classification of the order Caudovirales (Adriaenssens et al., 2020). As a result, phage 156 was reclassified as a member of the family *Herelleviridae*, subfamily *Brockvirinae*, genus *Kochikohdavirus*. Phage vB_EfaH_149 should be classified within the same genus given the homology found with phage 156 and other members of this genus (Figure 3). Similarly, phages Q69 and vb_EfaS_159 might be proposed new members of the genus *Efquatrovirus*. Based on its morphology, phage Q69 was previously designated as an unclassified *Siphoviridae* phage (Ladero et al., 2016a), but the determination of its genome sequence and its observed similarity with the genomes of other members of that genus, suggest it should be reclassified. Phage vB_EfaS_140 was found to be closely related to the *Enterococcus*-infecting *Siphoviridae* phages that possess a large genome of >80 kbp, while the majority of phage genomes belonging to this group are around 30–40 kbp long or shorter. Further investigation into the taxonomy and relationship of these phages with other large-genome *Siphoviridae* phages should be performed before they are assigned a taxonomic classification.

*Enterococcus*-infecting phages are traditionally screened in sewage water attending to the consideration of *Enterococcus* as member of the fecal microbiota. However, the members of *Enterococcus* genus are found in many environments, and are particularly common in foods of animal origin such as cheese. The phages described in the present work were distributed at different positions within the phylogenetic tree, and interspaced with phages isolated from other environments. Moreover, most were shown able to infect *E. faecalis* strains of non-dairy origin, including human (mostly fecal) and multiresistant strains from clinical environments. Genome analysis revealed the absence of virulence-related and antibiotic resistance genes in the studied genomes – essential safety features for their use in food or health applications. In summary, the present results increase the number of phages that might be used in cocktails to control *E. faecalis* in fermented foods, in which it may cause the accumulation of BAs, and perhaps in the clinical setting to fight against multiresistant enterococci.

## Data Availability Statement

The datasets presented in this study can be found in online repositories. The names of the repository/repositories and accession number(s) can be found below: https://www.ncbi.nlm.nih.gov/genbank/, CAJCJZ010000002.1; https://www.ncbi.nlm.nih.gov/genbank/, CAJDKF010000002.1; https://www.ncbi.nlm.nih.gov/genbank/, CAJDJX010000002.1; https://www.ncbi.nlm.nih.gov/genbank/, CAJDJZ010000002.1.

## Author Contributions

BR and VL designed and carried out some of the experiments and drafted the manuscript. ES-L and NM participated in the screening and phage characterization. BR, VL, and MF participated in the design of the study and helped write the manuscript. MA provided the general concept and supervised the work and the writing of the manuscript. All authors contributed to the discussions surrounding the work and approved the final version of the manuscript.

## Conflict of Interest

The authors declare that the research was conducted in the absence of any commercial or financial relationships that could be construed as a potential conflict of interest.

## Footnotes

1 http://cab.spbu.ru/software/spades/

2 http://rast.nmpdr.org/rast.cgi

3 http://blast.ncbi.nlm.nih.gov

4 https://mafft.cbrc.jp/alignment/server/

5 https://itol.embl.de/

## References

[B1] Adriaenssens E. M. Sullivan M. B. Knezevic P. van Zyl L. J. Sarkar B. L. Dutilh B. E. (2020). Taxonomy of prokaryotic viruses: 2018-2019 update from the ICTV Bacterial and Archaeal Viruses Subcommittee. *Arch. Virol.* 165 1253–1260. 10.1007/s00705-020-04577-8 32162068 PMC13502909

[B2] Altschul S. F. Madden T. L. Schaffer A. A. Zhang J. Zhang Z. Miller W. (1997). Gapped BLAST and PSI-BLAST: a new generation of protein database search programs. *Nucl. Acids Res.* 25 3389–3402. 10.1093/nar/25.17.3389 9254694 PMC146917

[B3] Aziz R. K. Bartels D. Best A. A. DeJongh M. Disz T. Edwards R. A. (2008). The RAST Server: rapid annotations using subsystems technology. *BMC Genom.* 9:75. 10.1186/1471-2164-9-75 18261238 PMC2265698

[B4] Bankevich A. Nurk S. Antipov D. Gurevich A. A. Dvorkin M. Kulikov A. S. (2012). SPAdes: a new genome assembly algorithm and its applications to single-cell sequencing. *J. Comput. Biol.* 19 455–477. 10.1089/cmb.2012.0021 22506599 PMC3342519

[B5] Barylski J. Kropinski A. M. Alikhan N.-F. Adriaenssens E. M. Ictv Report Consortium. (2020). ICTV Virus Taxonomy Profile: *herelleviridae*. *J. Gen. Virol.* 101 362–363. 10.1099/jgv.0.001392 32022658 PMC7414437

[B6] Beresford T. Williams A. (2004). “The microbiology of cheese ripening,” in *Cheese Chemistry, Physics and Microbiology*, eds Fox P. F. McSweeney P. L. H. Cogan T. M. Guinee T. P. (Amsterdam: Elsevier), 287–317.

[B7] Binetti A. G. del Rio B. Martin M. C. Alvarez M. A. (2005). Detection and characterization of *Streptococcus thermophilus* bacteriophages by use of the antireceptor gene sequence. *Appl. Environ. Microbiol.* 71 6096–6103. 10.1128/Aem.71.10.6096-6103.2005 16204526 PMC1265960

[B8] Bolocan A. S. Upadrasta A. Bettio P. H. A. Clooney A. G. Draper L. A. Ross R. P. (2019). Evaluation of phage therapy in the context of *Enterococcus faecalis* and its associated diseases. *Viruses* 11:366. 10.3390/v11040366 31010053 PMC6521178

[B9] Chan B. K. Abedon S. T. Loc-Carrillo C. (2013). Phage cocktails and the future of phage therapy. *Fut. Microbiol* 8 769–783. 10.2217/fmb.13.47 23701332

[B10] del Rio B. Redruello B. Ladero V. Cal S. Obaya A. J. Alvarez M. A. (2018). An altered gene expression profile in tyramine-exposed intestinal cell cultures supports the genotoxicity of this biogenic amine at dietary concentrations. *Sci. Rep.* 8:17038. 10.1038/s41598-018-35125-9 30451877 PMC6242974

[B11] del Rio B. Redruello B. Linares D. M. Ladero V. Ruas-Madiedo P. Fernandez M. (2019a). The biogenic amines putrescine and cadaverine show in vitro cytotoxicity at concentrations that can be found in foods. *Sci. Rep.* 9:120. 10.1038/s41598-018-36239-w 30644398 PMC6333923

[B12] del Rio B. Sanchez-Llana E. Redruello B. Magadan A. H. Fernandez M. Martin M. C. (2019b). *Enterococcus faecalis* bacteriophage 156 Is an effective biotechnological tool for reducing the presence of tyramine and putrescine in an experimental cheese model. *Front. Microbiol.* 10:566. 10.3389/fmicb.2019.00566 30949154 PMC6435515

[B13] EFSA (2011). Scientific Opinion on risk based control of biogenic amine formation in fermented foods. EFSA Panel on Biological Hazards (BIOHAZ). *EFSA J.* 9 2393–2486.

[B14] Fernandez L. Escobedo S. Gutierrez D. Portilla S. Martinez B. Garcia P. (2017). Bacteriophages in the dairy environment: from enemies to allies. *Antibiotics* 6:27. 10.3390/antibiotics6040027 29117107 PMC5745470

[B15] Fernández M. Linares D. M. del Río B. Ladero V. Alvarez M. A. (2007). HPLC quantification of biogenic amines in cheeses: correlation with PCR-detection of tyramine-producing microorganisms. *J. Dairy Res.* 74 276–282. 10.1017/S0022029907002488 17466118

[B16] Foulquie Moreno M. R. Sarantinopoulos P. Tsakalidou E. De Vuyst L. (2006). The role and application of enterococci in food and health. *Int. J. Food Microbiol.* 106 1–24. 10.1016/j.ijfoodmicro.2005.06.026 16216368

[B17] Garcia P. Martinez B. Obeso J. M. Rodriguez A. (2008). Bacteriophages and their application in food safety. *Lett. Appl. Microbiol.* 47 479–485. 10.1111/j.1472-765X.2008.02458.x 19120914

[B18] Giard J. C. Hartke A. Flahaut S. Benachour A. Boutibonnes P. Auffray Y. (1996). Starvation-induced multiresistance in *Enterococcus faecalis* JH2-2. *Curr. Microbiol.* 32 264–271. 10.1007/s002849900048 8857273

[B19] Giraffa G. (2003). Functionality of enterococci in dairy products. *Int. J. Food Microbiol.* 88 215–222. 10.1016/S0168-1605(03)00183-1 14596993

[B20] Gobbetti M. Di Cagno R. Calasso M. Neviani E. Fox P. F. De Angelis M. (2018). Drivers that establish and assembly the lactic acid bacteria biota in cheeses. *Trends Food Sci. Technol.* 78 244–254. 10.1016/j.tifs.2018.06.010

[B21] Gutierrez D. Martin-Platero A. Rodriguez A. Martinez-Bueno M. Garcia P. Martinez B. (2011). Typing of bacteriophages by randomly amplified polymorphic DNA (RAPD)-PCR to assess genetic diversity. *Fems Microbiol. Lett.* 322 90–97. 10.1111/j.1574-6968.2011.02342.x 21692832

[B22] Hesse S. Adhya S. (2019). Phage therapy in the twenty-first century: facing the decline of the antibiotic era; Is it finally time for the age of the phage? *Annu. Rev. Microbiol.* 73 155–174. 10.1146/annurev-micro-090817-62535 31185183

[B23] Katoh K. Rozewicki J. Yamada K. D. (2019). MAFFT online service: multiple sequence alignment, interactive sequence choice and visualization. *Br. Bioinform.* 20 1160–1166. 10.1093/bib/bbx108 28968734 PMC6781576

[B24] Khan H. Flint S. Yu P. L. (2010). Enterocins in food preservation. *Int. J. Food Microbiol.* 141 1–10. 10.1016/j.ijfoodmicro.2010.03.005 20399522

[B25] Kot W. Neve H. Heller K. J. Vogensen F. K. (2014). Bacteriophages of *leuconostoc*, *oenococcus*, and *weissella*. *Front. Microbiol.* 5:186. 10.3389/fmicb.2014.00186 24817864 PMC4009412

[B26] Ladero V. Calles-Enríquez M. Fernández M. Alvarez M. A. (2010a). Toxicological effects of dietary biogenic amines. *Curr. Nutr. Food Sci.* 6 145–156. 10.2174/157340110791233256 26958625

[B27] Ladero V. Canedo E. Perez M. Cruz Martin M. Fernandez M. Alvarez M. A. (2012a). Multiplex qPCR for the detection and quantification of putrescine-producing lactic acid bacteria in dairy products. *Food Control.* 27 307–313. 10.1016/j.foodcont.2012.03.024

[B28] Ladero V. Fernandez M. Alvarez M. A. (2009). Isolation and identification of tyramine-producing enterococci from human fecal samples. *Can. J. Microbiol.* 55 215–218. 10.1139/W08-133 19295656

[B29] Ladero V. Fernandez M. Calles-Enriquez M. Sanchez-Llana E. Canedo E. Martin M. C. (2012b). Is the production of the biogenic amines tyramine and putrescine a species-level trait in enterococci? *Food Microbiol.* 30 132–138. 10.1016/J.Fm.2011.12.016 22265293

[B30] Ladero V. Fernandez M. Cuesta I. Alvarez M. A. (2010b). Quantitative detection and identification of tyramine-producing enterococci and lactobacilli in cheese by multiplex qPCR. *Food Microbiol.* 27 933–939. 10.1016/J.Fm.2010.05.026 20688235

[B31] Ladero V. Gomez-Sordo C. Sanchez-Llana E. del Rio B. Redruello B. Fernandez M. (2016a). Q69 (an *E. faecalis*-Infecting bacteriophage) as a biocontrol agent for reducing tyramine in dairy products. *Front. Microbiol.* 7:445. 10.3389/Fmicb.2016.00445 27092117 PMC4820458

[B32] Ladero V. Linares D. M. Pérez M. del Rio B. Fernández M. Alvarez M. A. (2016b). *“Biogenic Amines in Dairy Products,” in Microbial Toxins in Dairy Products.* Hoboken, NJ: Wiley Blackwell, 94–131. 10.1002/9781118823095.ch4

[B33] Ladero V. Sanchez-Llana E. Fernandez M. Alvarez M. A. (2011). Survival of biogenic amine-producing dairy LAB strains at pasteurisation conditions. *Int. J. Food Sci. Technol.* 46 516–521. 10.1111/j.1365-2621.2010.02508.x

[B34] Letunic I. Bork P. (2016). Interactive tree of life (iTOL) v3: an online tool for the display and annotation of phylogenetic and other trees. *Nucl. Acids Res.* 44 W242–W245. 10.1093/nar/gkw290 27095192 PMC4987883

[B35] Linares D. M. Cruz Martin M. Ladero V. Alvarez M. A. Fernandez M. (2011). Biogenic amines in dairy products. *Crit. Rev. Food Sci. Nutr.* 51 691–703. 10.1080/10408398.2011.582813 21793728

[B36] Linares D. M. del Rio B. Redruello B. Ladero V. Martin M. C. Fernandez M. (2016). Comparative analysis of the in vitro cytotoxicity of the dietary biogenic amines tyramine and histamine. *Food Chem.* 197 658–663. 10.1016/j.foodchem.2015.11.013 26617000

[B37] Martínez B. García P. Gonzalez A. R. Piuri M. Raya R. R. (2016). “Bacteriophages of lactic acid bacteria and biotechnological tools,” in *Biotechnology of Lactic Acid Bacteria: Novel applications*, eds Mozzi F. Raya R. R. Vignolo G. (Chichester: JohnWiley & Sons), 100–119.

[B38] Moye Z. D. Woolston J. Sulakvelidze A. (2018). Bacteriophage applications for food production and processing. *Viruses* 10:205. 10.3390/v10040205 29671810 PMC5923499

[B39] Novella-Rodriguez S. Veciana-Nogues M. T. Trujillo-Mesa A. J. Vidal-Carou M. C. (2002). Profile of biogenic amines in goat cheese made from pasteurized and pressurized milks. *J. Food Sci.* 67 2940–2944.

[B40] Ogier J. C. Serror P. (2008). Safety assessment of dairy microorganisms: the *Enterococcus* genus. *Int. J. Food Microbiol.* 126 291–301. 10.1016/j.ijfoodmicro.2007.08.017 17889954

[B41] Paulsen I. T. Banerjei L. Myers G. S. Nelson K. E. Seshadri R. Read T. D. (2003). Role of mobile DNA in the evolution of vancomycin-resistant *Enterococcus faecalis*. *Science* 299 2071–2074. 10.1126/science.1080613 12663927

[B42] Perez M. Calles-Enriquez M. Nes I. Martin M. C. Fernandez M. Ladero V. (2015). Tyramine biosynthesis is transcriptionally induced at low pH and improves the fitness of *Enterococcus faecalis* in acidic environments. *Appl. Microbiol. Biotechnol.* 99 3547–3558. 10.1007/s00253-014-6301-7 25529314

[B43] Sillankorva S. M. Oliveira H. Azeredo J. (2012). Bacteriophages and their role in food safety. *Int. J. Microbiol.* 2012:863945. 10.1155/2012/863945 23316235 PMC3536431

[B44] Torres C. Alonso C. A. Ruiz-Ripa L. Leon-Sampedro R. Del Campo R. Coque T. M. (2018). Antimicrobial Resistance in *Enterococcus* spp. of animal origin. *Microbiol Spectr* 6:ARBA0032-2018. 10.1128/microbiolspec.ARBA-0032-2018 30051804 PMC11633606

[B45] Wunderlichová L. Buòková L. Koutnı M. Janèová P. Buòka F. (2014). Formation, degradation, and detoxification of putrescine by foodborne bacteria: a review. *Compr. Rev. Food Sci. Food Saf.* 13 1012–1030. 10.1111/1541-4337.12099

[B46] Yamaki S. Kawai Y. Yamazaki K. (2018). Biocontrol of *Morganella morganii* subsp. *morganii* and histamine accumulation in tuna meat by treatment with a lytic bacteriophage. *Food Sci. Technol. Res.* 24 329–337. 10.3136/fstr.24.329

