# Supplementary material for: Isolation and Characterization of Enterococcus faecalis-Infecting Bacteriophages From Different Cheese Types
Source: Front Microbiol. 2021 Jan 8;11:592172. doi: 10.3389/fmicb.2020.592172 (PMC7820071; doi:10.3389/fmicb.2020.592172)
Supplement: Supplementary file 1 [file Data_Sheet_1.ZIP › Supplementary Table 1 FINAL.docx]

**Supplementary Table 1**: Host range of the isolated *E. faecalis* phages. The origin of the bacterial strains is indicated (Type, dairy, meat, human or clinic).

| **Phage** | **Origin** | **Type** | **Dairy** | | | | | | | | | | | | | | **Meat** | | **Human** | | | | | | | | **Clinic** | |
| --- | --- | --- | --- | --- | --- | --- | --- | --- | --- | --- | --- | --- | --- | --- | --- | --- | --- | --- | --- | --- | --- | --- | --- | --- | --- | --- | --- | --- |
|  |  | **CECT481** | **15a** | **18a** | **19a** | **23a** | **28a** | **52c** | **54c** | **57c** | **63C** | **V61** | **V63** | **BA62** | **BA64** | **CECT4039** | **LMG12161** | **LMG20645** | **CECT795** | **CECT4176** | **HFS25** | **HFS57** | **HFS59** | **HFS62** | **HFS66** | **HFS69** | **V583** | **JH2-2** |
| vB_EfaS_140 | Gamoneu | **+** | **+** | **+** | **-** | **+** | **+** | **+** | **+** | **+** | **-** | **+** | **+** | **+** | **+** | **-** | **-** | **+** | **+** | **+** | **+** | **+** | **+** | **-** | **-** | **-** | **-** | **+** |
| 141 | Cabrales | **+** | **-** | **+** | **-** | **+** | **-** | **-** | **+** | **-** | **+** | **+** | **+** | **-** | **-** | **-** | **-** | **-** | **-** | **-** | **-** | **+** | **+** | **-** | **-** | **-** | **-** | **-** |
| 142 | Bofard | **-** | **-** | **-** | **-** | **-** | **-** | **-** | **-** | **-** | **-** | **-** | **-** | **-** | **-** | **-** | **-** | **-** | **+** | **-** | **-** | **-** | **-** | **-** | **-** | **-** | **-** | **-** |
| 143 | Emmental | **-** | **-** | **-** | **+** | **-** | **-** | **-** | **-** | **-** | **-** | **-** | **-** | **-** | **-** | **-** | **-** | **-** | **+** | **-** | **-** | **+** | **+** | **-** | **-** | **-** | **+** | **+** |
| 144 | Bofard | **-** | **-** | **-** | **+** | **-** | **-** | **-** | **-** | **-** | **-** | **-** | **-** | **+** | **-** | **-** | **-** | **-** | **+** | **+** | **-** | **+** | **+** | **-** | **-** | **-** | **+** | **-** |
| 145 | Bofard | **-** | **-** | **-** | **+** | **-** | **-** | **-** | **-** | **-** | **-** | **-** | **-** | **+** | **-** | **-** | **-** | **-** | **-** | **+** | **-** | **+** | **+** | **-** | **-** | **-** | **+** | **-** |
| 146 | Emmental | **-** | **-** | **-** | **+** | **-** | **-** | **-** | **-** | **-** | **-** | **-** | **-** | **-** | **-** | **-** | **-** | **-** | **-** | **+** | **-** | **-** | **-** | **-** | **-** | **-** | **+** | **+** |
| 147 | Zamorano | **-** | **-** | **-** | **+** | **-** | **-** | **-** | **-** | **-** | **-** | **-** | **-** | **-** | **-** | **-** | **-** | **-** | **-** | **+** | **-** | **+** | **-** | **-** | **-** | **-** | **-** | **+** |
| 148 | Zamorano | **+** | **-** | **+** | **+** | **+** | **+** | **+** | **+** | **+** | **+** | **+** | **+** | **+** | **+** | **-** | **+** | **-** | **-** | **-** | **-** | **+** | **+** | **-** | **-** | **-** | **+** | **-** |
| vB_EfaH_149 | Zamorano | **+** | **-** | **+** | **+** | **+** | **+** | **-** | **+** | **+** | **+** | **+** | **+** | **+** | **+** | **-** | **+** | **-** | **-** | **-** | **-** | **+** | **+** | **-** | **-** | **-** | **+** | **-** |
| 150 | Bofard | **-** | **-** | **-** | **-** | **-** | **+** | **-** | **+** | **-** | **-** | **+** | **+** | **+** | **+** | **-** | **-** | **-** | **-** | **-** | **-** | **+** | **+** | **-** | **-** | **-** | **+** | **-** |
| 151 | Bofard | **+** | **-** | **+** | **-** | **-** | **-** | **-** | **-** | **-** | **-** | **+** | **-** | **-** | **-** | **-** | **-** | **-** | **-** | **-** | **-** | **-** | **-** | **-** | **-** | **-** | **+** | **-** |
| 152 | Cabrales | **+** | **-** | **+** | **-** | **+** | **-** | **-** | **-** | **-** | **-** | **+** | **-** | **+** | **-** | **-** | **-** | **-** | **-** | **-** | **-** | **-** | **-** | **-** | **-** | **-** | **-** | **-** |
| 153 | Bofard | **+** | **-** | **+** | **+** | **+** | **-** | **-** | **-** | **-** | **+** | **-** | **-** | **-** | **-** | **-** | **-** | **-** | **-** | **-** |  | **+** | **+** | **-** | **-** | **-** | **-** | **-** |
| 155 | Cabrales | **+** | **-** | **-** | **-** | **-** | **-** | **-** | **-** | **-** | **-** | **-** | **-** | **-** | **-** | **-** | **-** | **-** | **-** | **-** | **-** | **-** | **-** | **-** | **-** | **-** | **-** | **-** |
| 156 | Sheep Cheese | **+** | **+** | **+** | **+** | **+** | **+** | **-** | **+** | **+** | **+** | **+** | **+** | **+** | **+** | **-** | **+** | **-** | **+** | **-** | **-** | **+** | **+** | **-** | **-** | **-** | **+** | **+** |
| 157 | Sheep Cheese | **+** | **+** | **+** | **+** | **+** | **-** | **+** | **+** | **+** | **+** | **+** | **+** | **+** | **+** | **-** | **+** | **-** | **+** | **-** | **-** | **+** | **+** | **-** | **-** | **-** | **+** | **+** |
| 158 | Cabrales | **+** | **-** | **-** | **+** | **+** | **+** | **+** | **-** | **+** | **+** | **-** | **+** | **-** | **-** | **-** | **-** | **-** | **-** | **-** | **-** | **+** | **+** | **-** | **-** | **-** | **-** | **-** |
| vB_EfaS_159 | Cabrales | **+** | **-** | **+** | **+** | **+** | **-** | **+** | **-** | **+** | **-** | **-** | **+** | **-** | **-** | **-** | **-** | **-** | **-** | **-** | **-** | **+** | **+** | **-** | **-** | **-** | **-** | **-** |
| 160 | Cabrales | **+** | **-** | **+** | **+** | **-** | **-** | **+** | **-** | **+** | **+** | **-** | **+** | **-** | **-** | **-** | **-** | **-** | **-** | **-** | **-** | **+** | **+** | **-** | **-** | **-** | **-** | **-** |
| 161 | Cabrales | **+** | **-** | **+** | **+** | **+** | **+** | **+** | **-** | **+** | **+** | **-** | **-** | **-** | **-** | **-** | **-** | **-** | **-** | **-** |  | **-** | **-** | **-** | **-** | **-** | **-** | **-** |
| Q69 | Goat Cheese | **+** | **-** | **+** | **+** | **+** | **-** | **+** | **-** | **+** | **+** | **-** | **-** | **-** | **-** | **-** | **-** | **-** | **+** | **-** | **-** | **+** | **+** | **-** | **-** | **-** | **-** | **-** |

+: plaques detected; -: absence of plaques;
