# Supplementary material for: Isolation and Characterization of Enterococcus faecalis-Infecting Bacteriophages From Different Cheese Types
Source: Front Microbiol. 2021 Jan 8;11:592172. doi: 10.3389/fmicb.2020.592172 (PMC7820071; doi:10.3389/fmicb.2020.592172)
Supplement: Supplementary file 1 [file Data_Sheet_1.ZIP › Supplementary Table 2 FINAL.docx]

**Supplementary Table 2**: Complete bacteriophage genomes used for the construction of the phylogenetic trees. Each phage's family, subfamily and genus is indicated (if known), along with its origin, the genome size in bp, and the corresponding accession number. UC: Unclassified; NI: Not indicated.

| **Name** | **Family/**  **Subfamily** | **Genus** | **Origin** | **Genome Size** | **Accession** |
| --- | --- | --- | --- | --- | --- |
| 156 | *Herelleviridae/Brockvirinae* | *Kochikohdavirus* | Cheese | 141133 | LR031359.1 |
| ECP3 | *Herelleviridae/Brockvirinae* | *Kochikohdavirus* | NI | 145518 | KJ801817 |
| EFLK1 | *Herelleviridae/Brockvirinae* | *Kochikohdavirus* | Sewage | 130952 | KR049063 |
| PBEF129 | *Herelleviridae/Brockvirinae* | *Kochikohdavirus* | NI | 144230 | MN854830 |
| phiEF24C | *Herelleviridae/Brockvirinae* | *Kochikohdavirus* | NI | 142072 | AP009390 |
| vB_EfaH_EF1TV | *Herelleviridae/Brockvirinae* | *Kochikohdavirus* | Hospital wastewater | 143507 | MK268686 |
| vB_EfaM_Ef2.1 | *Herelleviridae/Brockvirinae* | *Kochikohdavirus* | Sewage water | 140938 | MK693030 |
| vB_EfaM_Ef2.3 | *Herelleviridae/Brockvirinae* | *Kochikohdavirus* | Sewage water | 147289 | MK721192 |
| EFDG1 | *Herelleviridae/Brockvirinae* | UC | Sewage | 147589 | KP339049 |
| EfsSzw-1 | *Herelleviridae/Brockvirinae* | UC | Sewage water | 150272 | MH791397 |
| PEf771 | *Herelleviridae/Brockvirinae* | UC | Water | 151052 | MN241318.1 |
| vB_OCPT_Ben | *Herelleviridae/Brockvirinae* | UC | Sewage | 151985 | MN027503 |
| vB_EfaH_149 | *Herelleviridae/*UC | UC | Cheese | 142215 | CAJDJZ010000002.1 |
| EF62phi | *Podoviridae/Picovirinae* | UC | Human | 30505 | CP002495 |
| vB_EfaP_Ef6.2 | *Podoviridae/Picovirinae* | UC | Sewage water | 17966 | MK721188 |
| vB_EfaP_Ef6.3 | *Podoviridae/Picovirinae* | UC | Sewage water | 18136 | MK721196 |
| vB_EfaP_Ef7.2 | *Podoviridae/Picovirinae* | UC | Sewage water | 18737 | MK721183 |
| vB_EfaP_Ef7.3 | *Podoviridae/Picovirinae* | UC | Sewage water | 18818 | MK721184 |
| vB_EfaP_Ef7.4 | *Podoviridae/Picovirinae* | UC | Sewage water | 18415 | MK721198 |
| vB_EfaP_Efmus1 | *Podoviridae/Picovirinae* | UC | Sewage water | 17927 | MK721195 |
| vB_EfaP_Efmus2 | *Podoviridae/Picovirinae* | UC | Sewage water | 18366 | MK721197 |
| vB_EfaP_Efmus3 | *Podoviridae/Picovirinae* | UC | Sewage water | 18286 | MK721185 |
| vB_EfaP_Efmus4 | *Podoviridae/Picovirinae* | UC | Sewage water | 18186 | MK721193 |
| vB_EfaP_IME195 | *Podoviridae/Picovirinae* | UC | Hospital sewage | 18607 | NC_028693 |
| AUEF3 | *Siphoviridae* | *Efquatrovirus* | Sewage | 41257 | KJ127304 |
| EfaCPT1 | *Siphoviridae* | *Efquatrovirus* | Sewage | 40923 | JX193904 |
| heks | *Siphoviridae* | *Efquatrovirus* | Wastewater | 39708 | MT119359 |
| IME_EF3 | *Siphoviridae* | *Efquatrovirus* | Sewage | 41687 | KF728385 |
| LY0322 | *Siphoviridae* | *Efquatrovirus* | NI | 40934 | MH193369 |
| Nonaheksakonda | *Siphoviridae* | *Efquatrovirus* | Wastewater | 41994 | MK125140 |
| phiNASRA1 | *Siphoviridae* | *Efquatrovirus* | NI | 40139 | MG264739 |
| phiSHEF2 | *Siphoviridae* | *Efquatrovirus* | Wastewater | 41712 | MF678788 |
| phiSHEF4 | *Siphoviridae* | *Efquatrovirus* | Wastewater | 41081 | MF678789 |
| phiSHEF5 | *Siphoviridae* | *Efquatrovirus* | Wastewater | 41598 | MF678790 |
| PMBT2 | *Siphoviridae* | *Efquatrovirus* | Sewage water | 41489 | MG708276 |
| SANTOR1 | *Siphoviridae* | *Efquatrovirus* | Sewage water | 37933 | KX284704 |
| vB_EfaS_AL2 | *Siphoviridae* | *Efquatrovirus* | Wastewater | 40836 | MH203384 |
| vB_EfaS_AL3 | *Siphoviridae* | *Efquatrovirus* | Wastewater | 40789 | MH203383 |
| vB_EfaS_Ef5.1 | *Siphoviridae* | *Efquatrovirus* | Sewage | 41141 | MK721199 |
| vB_EfaS_Ef5.2 | *Siphoviridae* | *Efquatrovirus* | Sewage | 41418 | MK721186 |
| vB_EfaS_Ef5.3 | *Siphoviridae* | *Efquatrovirus* | Sewage | 39115 | MK721200 |
| vB_EfaS_Ef5.4 | *Siphoviridae* | *Efquatrovirus* | Sewage | 40685 | MK721191 |
| vB_EfaS_Ef6.1 | *Siphoviridae* | *Efquatrovirus* | Sewage | 40429 | MK721187 |
| vB_EfaS_Ef6.4 | *Siphoviridae* | *Efquatrovirus* | Sewage | 41133 | MK721190 |
| vB_EfaS_IME196 | *Siphoviridae* | *Efquatrovirus* | Hospital sewage | 38886 | NC_028990 |
| vB_EfaS_LM99 | *Siphoviridae* | *Efquatrovirus* | Sewage | 40203 | MH355583 |
| vB_EfaS_Max | *Siphoviridae* | *Efquatrovirus* | Sewage | 40975 | MK360024 |
| phiFL1A | *Siphoviridae* | *Phifelvirus* | NI | 38764 | NC_013646 |
| phiFL1B | *Siphoviridae* | *Phifelvirus* | NI | 38989 | GQ478082 |
| phiFL1C | *Siphoviridae* | *Phifelvirus* | NI | 38721 | GQ478083 |
| phiFL2A | *Siphoviridae* | *Phifelvirus* | NI | 36270 | GQ478084 |
| phiFL2B | *Siphoviridae* | *Phifelvirus* | NI | 36826 | GQ478085 |
| phiFL3A | *Siphoviridae* | *Phifelvirus* | NI | 39576 | NC_013648 |
| phiFL3B | *Siphoviridae* | *Phifelvirus* | NI | 40275 | GQ478087 |
| BC-611 | *Siphoviridae* | *Saphexavirus* | NI | 53996 | AB712291 |
| EF-P10 | *Siphoviridae* | *Saphexavirus* | Sewage | 57408 | KY472224 |
| EF-P29 | *Siphoviridae* | *Saphexavirus* | Sewage | 58984 | KY303907 |
| EfsWh-1 | *Siphoviridae* | *Saphexavirus* | Freshwater | 58036 | MH791415 |
| Entf1 | *Siphoviridae* | *Saphexavirus* | Sewage | 58938 | MK800154 |
| IME_EF1 | *Siphoviridae* | *Saphexavirus* | Sewage | 57081 | KF192053 |
| SAP6 | *Siphoviridae* | *Saphexavirus* | NI | 58619 | JF731128 |
| vB_EfaS_EF1c55 | *Siphoviridae* | *Saphexavirus* | NI | 55876 | MN103542 |
| vB_EfaS_Ef2.2 | *Siphoviridae* | *Saphexavirus* | Sewage | 58400 | MK721189 |
| vB_EfaS_Ef7.1 | *Siphoviridae* | *Saphexavirus* | Sewage | 58018 | MK721194 |
| vB_EfaS_HEf13 | *Siphoviridae* | *Saphexavirus* | NI | 57811 | MH618488 |
| vB_EfaS_IME198 | *Siphoviridae* | *Saphexavirus* | Hospital sewage | 58000 | NC_029016 |
| vB_EfaS_PHB08 | *Siphoviridae* | *Saphexavirus* | NI | 55244 | MK570225 |
| VD13 | *Siphoviridae* | *Saphexavirus* | NI | 55113 | KJ094032 |
| vB_EfaS_140 | *Siphoviridae* | UC | Cheese | 85454 | CAJCJZ010000002.1 |
| vB_EfaS_159 | *Siphoviridae* | UC | Cheese | 41718 | CAJDKF010000002.1 |
| EFAP-1 | *Siphoviridae* | UC | NI | 21115 | FJ792813 |
| EFC-1 | *Siphoviridae* | UC | NI | 40286 | KJ608188 |
| EFP1 | *Siphoviridae* | UC | NI | 37561 | MN995824 |
| EFRM31 | *Siphoviridae* | UC | NI | 16945 | GU815339 |
| nattely | *Siphoviridae* | UC | Wastewater | 85669 | MT119360 |
| phiEf11 | *Siphoviridae* | UC | NI | 42822 | GQ452243 |
| phiFL4A | *Siphoviridae* | UC | NI | 37856 | NC_013644 |
| Q69 | *Siphoviridae* | UC | Cheese | 42141 | CAJDJX010000002.1 |
| vB_EfaS_DELF1 | *Siphoviridae* | UC | NI | 40248 | LC513943 |
| vB_EfaS_IME197 | *Siphoviridae* | UC | NI | 41098 | KT945994 |
| vipetofem | *Siphoviridae* | UC | Wastewater | 85371 | MT119361 |
