# Supplementary material for: Isolation and Characterization of Enterococcus faecalis-Infecting Bacteriophages From Different Cheese Types
Source: Front Microbiol. 2021 Jan 8;11:592172. doi: 10.3389/fmicb.2020.592172 (PMC7820071; doi:10.3389/fmicb.2020.592172)
Supplement: Supplementary file 1 [file Data_Sheet_1.ZIP › Supplementary Table 3 FINAL.docx]

**Supplementary Table 3a:** Features of *Enterococcus faecalis* bacteriophage vB_EfaH_149. The CDS gene number, and gene position in the phage vB_EfaS_149 genome are shown, as are the predicted length and function of the encoded products. The position of the tRNA found in the genome are also indicated.

| Gene | Start (bp) | Stop (bp) | Length (aa) | Predicted function |
| --- | --- | --- | --- | --- |
| CDS_1 | 113 | 382 | 89 | hypothetical protein |
| CDS_2 | 685 | 1113 | 142 | hypothetical protein |
| CDS_3 | 1113 | 1481 | 122 | Phage terminase, large subunit |
| CDS_4 | 1745 | 2704 | 319 | putative group I intron protein |
| CDS_5 | 2768 | 3061 | 97 | Phage terminase, large subunit |
| CDS_6 | 3352 | 4281 | 309 | hypothetical protein |
| CDS_7 | 4355 | 5539 | 394 | Phage terminase, large subunit |
| CDS_8 | 5639 | 6427 | 262 | hypothetical protein |
| CDS_9 | 6616 | 7248 | 210 | hypothetical protein |
| CDS_10 | 7256 | 7582 | 108 | hypothetical protein |
| CDS_11 | 7676 | 8545 | 289 | Phage lysin, N-acetylmuramoyl-L-alanine amidase |
| CDS_12 | 8712 | 9380 | 222 | peptidoglycan-binding LysM |
| CDS_13 | 9522 | 9866 | 114 | hypothetical protein |
| CDS_14 | 9881 | 11605 | 574 | portal protein |
| CDS_15 | 11612 | 11725 | 37 | hypothetical protein |
| CDS_16 | 11712 | 12503 | 263 | putative prohead protease |
| CDS_17 | 12510 | 13475 | 321 | hypothetical protein |
| CDS_18 | 13616 | 15010 | 464 | Phage major capsid protein |
| CDS_19 | 15115 | 15378 | 87 | hypothetical protein |
| CDS_20 | 15391 | 16290 | 299 | hypothetical protein |
| CDS_21 | 16309 | 17178 | 289 | hypothetical protein |
| CDS_22 | 17171 | 17794 | 207 | hypothetical protein |
| CDS_23 | 17798 | 18643 | 281 | hypothetical protein |
| CDS_24 | 18643 | 18882 | 79 | hypothetical protein |
| CDS_25 | 18886 | 20595 | 569 | Phage major tail sheath |
| CDS_26 | 20656 | 21078 | 140 | Phage structural protein |
| CDS_27 | 21170 | 22267 | 365 | hypothetical protein |
| CDS_28 | 22264 | 22410 | 48 | hypothetical protein |
| CDS_29 | 22547 | 23020 | 157 | hypothetical protein |
| CDS_30 | 23088 | 23663 | 191 | hypothetical protein |
| CDS_31 | 23708 | 27364 | 1218 | Phage tail length tape-measure protein |
| CDS_32 | 27403 | 30588 | 1061 | Secretory antigen SsaA-like protein |
| CDS_33 | 30683 | 36151 | 1822 | putative tail fiber |
| CDS_34 | 36374 | 38662 | 762 | putative minor structural protein |
| CDS_35 | 38656 | 39381 | 241 | hypothetical protein |
| CDS_36 | 39413 | 39553 | 46 | hypothetical protein |
| CDS_37 | 39634 | 40338 | 234 | hypothetical protein |
| CDS_38 | 40425 | 41114 | 229 | hypothetical protein |
| CDS_39 | 41118 | 41654 | 178 | hypothetical protein |
| CDS_40 | 41641 | 42345 | 234 | putative baseplate |
| CDS_41 | 42361 | 43413 | 350 | putative baseplate J-like protein |
| CDS_42 | 43432 | 44832 | 466 | hypothetical protein |
| CDS_43 | 44938 | 45483 | 181 | hypothetical protein |
| CDS_44 | 45498 | 48962 | 1154 | putative adsorption associated tail protein |
| CDS_45 | 49044 | 50006 | 320 | hypothetical protein |
| CDS_46 | 50084 | 50290 | 68 | hypothetical protein |
| CDS_47 | 50545 | 53589 | 1014 | DNA helicase, phage-associated |
| CDS_48 | 53617 | 55245 | 542 | hypothetical protein |
| CDS_49 | 55278 | 57812 | 844 | DNA helicase, phage-associated |
| CDS_50 | 57812 | 58867 | 351 | Phage recombination exonuclease |
| CDS_51 | 58983 | 60866 | 627 | Phage recombination related exonuclease |
| CDS_52 | 60884 | 61549 | 221 | hypothetical protein |
| CDS_53 | 61550 | 62608 | 352 | DNA primase |
| CDS_54 | 62625 | 63257 | 210 | hypothetical protein |
| CDS_55 | 63286 | 64170 | 294 | Deoxyuridine 5'-triphosphate nucleotidohydrolase |
| CDS_56 | 64173 | 64403 | 76 | hypothetical protein |
| CDS_57 | 64405 | 64713 | 102 | hypothetical protein |
| CDS_58 | 64700 | 65011 | 103 | hypothetical protein |
| CDS_59 | 65004 | 65375 | 123 | hypothetical protein |
| CDS_60 | 65395 | 66063 | 222 | putative resolvase |
| CDS_61 | 66065 | 66364 | 99 | hypothetical protein |
| CDS_62 | 66370 | 66849 | 159 | hypothetical protein |
| CDS_63 | 66942 | 67736 | 264 | hypothetical protein |
| CDS_64 | 67798 | 68040 | 80 | Phage integration host factor |
| CDS_65 | 68130 | 70532 | 800 | DNA polymerase I, phage-associated |
| CDS_66 | 70702 | 71364 | 220 | putative HMH homimg endonuclease |
| CDS_67 | 71693 | 72217 | 174 | DNA polymerase I, phage-associated |
| CDS_68 | 72320 | 72862 | 180 | hypothetical protein |
| CDS_69 | 72919 | 74208 | 429 | hypothetical protein |
| CDS_70 | 74293 | 75540 | 415 | Phage recombinase |
| CDS_71 | 75594 | 75980 | 128 | hypothetical protein |
| CDS_72 | 75973 | 76587 | 204 | hypothetical protein |
| CDS_73 | 76648 | 76923 | 91 | hypothetical protein |
| CDS_74 | 76971 | 77915 | 314 | hypothetical protein |
| CDS_75 | 77948 | 78391 | 147 | hypothetical protein |
| CDS_76 | 78497 | 78793 | 98 | hypothetical protein |
| CDS_77 | 78798 | 79751 | 317 | hypothetical protein |
| CDS_78 | 79805 | 81088 | 427 | hypothetical protein |
| CDS_79 | 81100 | 81474 | 124 | hypothetical protein |
| CDS_80 | 81513 | 82133 | 206 | hypothetical protein |
| CDS_81 | 82133 | 82873 | 246 | hypothetical protein |
| CDS_82 | 82863 | 83369 | 168 | hypothetical protein |
| CDS_83 | 83383 | 84240 | 285 | hypothetical protein |
| CDS_84 | 84344 | 85189 | 281 | Thioredoxin, phage-associated |
| CDS_85 | 85182 | 86810 | 542 | hypothetical protein |
| CDS_86 | 87129 | 87818 | 229 | hypothetical protein |
| CDS_87 | 87829 | 88296 | 155 | hypothetical protein |
| CDS_88 | 88396 | 90675 | 759 | hypothetical protein |
| CDS_89 | 90960 | 91466 | 168 | hypothetical protein |
| CDS_90 | 91541 | 91711 | 56 | hypothetical protein |
| CDS_91 | 91701 | 91973 | 90 | hypothetical protein |
| CDS_92 | 92081 | 92332 | 83 | hypothetical protein |
| CDS_93 | 92344 | 92631 | 95 | hypothetical protein |
| CDS_94 | 92634 | 93422 | 262 | hypothetical protein |
| CDS_95 | 93504 | 94577 | 357 | LPXTG-motif cell wall anchor domain protein |
| CDS_96 | 94703 | 95005 | 100 | hypothetical protein |
| CDS_97 | 95007 | 95309 | 100 | hypothetical protein |
| CDS_98 | 95312 | 95605 | 97 | hypothetical protein |
| CDS_99 | 95602 | 95781 | 59 | hypothetical protein |
| CDS_100 | 95794 | 96159 | 121 | hypothetical protein |
| CDS_101 | 96186 | 96563 | 125 | hypothetical protein |
| CDS_102 | 96556 | 96786 | 76 | hypothetical protein |
| CDS_103 | 96976 | 97164 | 62 | hypothetical protein |
| CDS_104 | 97161 | 97418 | 85 | hypothetical protein |
| CDS_105 | 97424 | 98017 | 197 | hypothetical protein |
| CDS_106 | 98059 | 98901 | 280 | hypothetical protein |
| CDS_107 | 98914 | 99216 | 100 | hypothetical protein |
| CDS_108 | 99217 | 99846 | 209 | hypothetical protein |
| CDS_109 | 99925 | 100167 | 80 | hypothetical protein |
| CDS_110 | 100184 | 100324 | 46 | hypothetical protein |
| CDS_111 | 100337 | 100669 | 110 | hypothetical protein |
| CDS_112 | 100734 | 101390 | 218 | hypothetical protein |
| CDS_113 | 101387 | 101830 | 147 | hypothetical protein |
| CDS_114 | 101883 | 102443 | 186 | hypothetical protein |
| CDS_115 | 102661 | 102897 | 78 | hypothetical protein |
| CDS_116 | 102959 | 103147 | 62 | hypothetical protein |
| CDS_117 | 103141 | 103254 | 37 | hypothetical protein |
| CDS_118 | 103361 | 103603 | 80 | hypothetical protein |
| CDS_119 | 103777 | 104226 | 149 | hypothetical protein |
| CDS_120 | 104676 | 104975 | 99 | hypothetical protein |
| CDS_121 | 105053 | 105376 | 107 | hypothetical protein |
| CDS_122 | 105473 | 105613 | 46 | hypothetical protein |
| CDS_123 | 106167 | 106349 | 60 | hypothetical protein |
| CDS_124 | 106431 | 106772 | 113 | hypothetical protein |
| CDS_125 | 106830 | 106964 | 44 | hypothetical protein |
| CDS_126 | 107107 | 107334 | 75 | hypothetical protein |
| CDS_127 | 107418 | 107693 | 91 | hypothetical protein |
| CDS_128 | 108108 | 108311 | 67 | hypothetical protein |
| CDS_129 | 108353 | 108667 | 104 | hypothetical protein |
| CDS_130 | 108721 | 109032 | 103 | hypothetical protein |
| CDS_131 | 109818 | 110009 | 63 | hypothetical protein |
| CDS_132 | 110053 | 110277 | 74 | hypothetical protein |
| CDS_133 | 110291 | 110542 | 83 | hypothetical protein |
| CDS_134 | 110543 | 110923 | 126 | hypothetical protein |
| CDS_135 | 111159 | 111356 | 65 | hypothetical protein |
| CDS_136 | 111356 | 111871 | 171 | hypothetical protein |
| CDS_137 | 111884 | 112132 | 82 | hypothetical protein |
| CDS_138 | 112145 | 112687 | 180 | hypothetical protein |
| CDS_139 | 112684 | 113076 | 130 | hypothetical protein |
| CDS_140 | 113073 | 113429 | 118 | hypothetical protein |
| CDS_141 | 113433 | 113603 | 56 | hypothetical protein |
| CDS_142 | 113591 | 114091 | 166 | hypothetical protein |
| CDS_143 | 114094 | 114558 | 154 | hypothetical protein |
| CDS_144 | 114555 | 115025 | 156 | hypothetical protein |
| CDS_145 | 115227 | 115592 | 121 | hypothetical protein |
| CDS_146 | 115589 | 115768 | 59 | hypothetical protein |
| CDS_147 | 115782 | 116300 | 172 | hypothetical protein |
| CDS_148 | 116301 | 116774 | 157 | hypothetical protein |
| CDS_149 | 116771 | 117478 | 235 | hypothetical protein |
| CDS_150 | 117507 | 117947 | 146 | hypothetical protein |
| CDS_151 | 117997 | 118185 | 62 | hypothetical protein |
| CDS_152 | 118182 | 118643 | 153 | hypothetical protein |
| CDS_153 | 118657 | 118833 | 58 | hypothetical protein |
| CDS_154 | 118830 | 119027 | 65 | hypothetical protein |
| CDS_155 | 119024 | 119461 | 145 | hypothetical protein |
| CDS_156 | 119552 | 120292 | 246 | putative serine/threonine protein phosphatase |
| CDS_157 | 120289 | 120456 | 55 | hypothetical protein |
| CDS_158 | 121020 | 121631 | 203 | putative phosphoesterase |
| CDS_159 | 121644 | 121994 | 116 | hypothetical protein |
| CDS_160 | 121991 | 122107 | 38 | hypothetical protein |
| CDS_161 | 122121 | 122333 | 70 | hypothetical protein |
| CDS_162 | 122414 | 122827 | 137 | hypothetical protein |
| CDS_163 | 122832 | 123215 | 127 | hypothetical protein |
| CDS_164 | 123217 | 123492 | 91 | hypothetical protein |
| CDS_165 | 123489 | 123683 | 64 | hypothetical protein |
| CDS_166 | 123839 | 124012 | 57 | hypothetical protein |
| CDS_167 | 124002 | 124346 | 114 | hypothetical protein |
| CDS_168 | 124346 | 124600 | 84 | hypothetical protein |
| CDS_169 | 124581 | 124835 | 84 | hypothetical protein |
| CDS_170 | 124846 | 125181 | 111 | hypothetical protein |
| CDS_171 | 125156 | 125593 | 145 | hypothetical protein |
| CDS_172 | 125609 | 126070 | 153 | hypothetical protein |
| CDS_173 | 126063 | 126290 | 75 | hypothetical protein |
| CDS_174 | 126278 | 126709 | 143 | hypothetical protein |
| CDS_175 | 126712 | 126942 | 76 | hypothetical protein |
| CDS_176 | 126939 | 127370 | 143 | hypothetical protein |
| CDS_177 | 127374 | 127526 | 50 | hypothetical protein |
| CDS_178 | 127526 | 128473 | 315 | putative thymidylate synthase |
| CDS_179 | 128660 | 128809 | 49 | hypothetical protein |
| CDS_180 | 128809 | 129666 | 285 | hypothetical protein |
| CDS_181 | 129827 | 130156 | 109 | putative ribonucleotide reductase |
| CDS_182 | 130524 | 131078 | 184 | hypothetical protein |
| CDS_183 | 131268 | 131855 | 195 | putative ribonucleotide reductase |
| CDS_184 | 131868 | 134018 | 716 | putative ribonucleotide reductase |
| CDS_185 | 134021 | 134263 | 80 | hypothetical protein |
| CDS_186 | 134390 | 134656 | 88 | hypothetical protein |
| CDS_187 | 134662 | 134967 | 101 | hypothetical protein |
| CDS_188 | 135058 | 135288 | 76 | hypothetical protein |
| CDS_189 | 135352 | 135576 | 74 | hypothetical protein |
| CDS_190 | 135687 | 135971 | 94 | hypothetical protein |
| tRNA_1 | 136023 | 136094 | - | tRNA-Thr |
| CDS_191 | 136119 | 137210 | 363 | hypothetical protein |
| tRNA_2 | 137581 | 137654 | - | tRNA-Met |
| tRNA_3 | 137713 | 137786 | - | tRNA-Pro |
| tRNA_4 | 138736 | 138809 | - | tRNA-Arg |
| tRNA_5 | 139292 | 139363 | - | tRNA-Trp |
| tRNA_6 | 139619 | 139691 | - | tRNA-Pseudo-GTC |
| CDS_192 | 141170 | 141508 | 112 | hypothetical protein |
| CDS_193 | 141572 | 141916 | 114 | hypothetical protein |

**Supplementary Table 3b:** Features of *Enterococcus faecalis* bacteriophage Q69. The CDS gene number, and gene position in the phage Q69 genome are shown, as are the predicted length and function of the encoded products.

| Gene | Start (bp) | Stop (bp) | Length (aa) | Predicted function |
| --- | --- | --- | --- | --- |
| CDS_1 | 109 | 309 | 66 | hypothetical protein |
| CDS_2 | 309 | 557 | 82 | hypothetical protein |
| CDS_3 | 557 | 952 | 131 | HNH endocuclease |
| CDS_4 | 1218 | 1391 | 57 | hypothetical protein |
| CDS_5 | 1406 | 1879 | 157 | Phage terminase, small subunit |
| CDS_6 | 2483 | 4210 | 575 | Phage terminase, large subunit |
| CDS_7 | 4299 | 4619 | 106 | hypothetical protein |
| CDS_8 | 4638 | 5150 | 170 | Mobile element-transposase |
| CDS_9 | 5192 | 5977 | 261 | Mobile element-Integrase |
| CDS_10 | 6079 | 6243 | 54 | hypothetical protein |
| CDS_11 | 6248 | 7426 | 392 | Phage portal protein |
| CDS_12 | 7401 | 7955 | 184 | Phage head maturation protease |
| CDS_13 | 8032 | 9252 | 406 | Major capsid protein |
| CDS_14 | 9378 | 9581 | 67 | Tail protein |
| CDS_15 | 9623 | 9916 | 97 | Head-Tail joining protein |
| CDS_16 | 9891 | 10223 | 110 | Head-Tail joining protein |
| CDS_17 | 10220 | 10627 | 135 | Head-Tail joining protein |
| CDS_18 | 10624 | 10989 | 121 | Head-Tail joining protein |
| CDS_19 | 11068 | 11634 | 188 | Phage major tail protein |
| CDS_20 | 11809 | 12141 | 110 | Putetive tail length tape-measure chaperon protein |
| CDS_21 | 12207 | 12380 | 57 | hypothetical protein |
| CDS_22 | 12380 | 16267 | 1295 | Phage tail length tape-measure protein |
| CDS_23 | 16349 | 18424 | 691 | Phage tail protein |
| CDS_24 | 18469 | 20697 | 742 | Phage tail protein |
| CDS_25 | 20877 | 21122 | 81 | putative phage tail host specificity protein J |
| CDS_26 | 21137 | 21370 | 77 | Phage holin |
| CDS_27 | 21373 | 22470 | 365 | Phage lysin, N-acetylmuramoyl-L-alanine amidase (EC 3.5.1.28) |
| CDS_28 | 22560 | 22787 | 75 | hypothetical protein |
| CDS_29 | 22788 | 23594 | 268 | hypothetical protein |
| CDS_30 | 23683 | 25974 | 763 | DNA polymerase B region |
| CDS_31 | 26010 | 26234 | 74 | hypothetical protein |
| CDS_32 | 26304 | 27008 | 234 | hypothetical protein |
| CDS_33 | 27086 | 27334 | 82 | hypothetical protein |
| CDS_34 | 27335 | 27631 | 98 | hypothetical protein |
| CDS_35 | 27632 | 28450 | 272 | hypothetical protein |
| CDS_36 | 28440 | 28622 | 60 | hypothetical protein |
| CDS_37 | 28612 | 28776 | 54 | hypothetical protein |
| CDS_38 | 28749 | 29528 | 259 | hypothetical protein |
| CDS_39 | 29539 | 30018 | 159 | putative HNH homing endonuclease-like protein |
| CDS_40 | 30015 | 30221 | 68 | hypothetical protein |
| CDS_41 | 30223 | 30498 | 91 | hypothetical protein |
| CDS_42 | 30470 | 30727 | 85 | hypothetical protein |
| CDS_43 | 30888 | 31097 | 69 | hypothetical protein |
| CDS_44 | 31097 | 31837 | 246 | Primase/polymerase |
| CDS_45 | 31849 | 32037 | 62 | hypothetical protein |
| CDS_46 | 32096 | 32272 | 58 | hypothetical protein |
| CDS_47 | 32269 | 33564 | 431 | Helicase |
| CDS_48 | 33557 | 33946 | 129 | hypothetical protein |
| CDS_49 | 33930 | 34166 | 78 | hypothetical protein |
| CDS_50 | 34170 | 34379 | 69 | hypothetical protein |
| CDS_51 | 34381 | 34542 | 53 | hypothetical protein |
| CDS_52 | 34544 | 34828 | 94 | hypothetical protein |
| CDS_53 | 34961 | 35422 | 153 | hypothetical protein |
| CDS_54 | 35497 | 35667 | 56 | hypothetical protein |
| CDS_55 | 35740 | 35928 | 62 | hypothetical protein |
| CDS_56 | 36018 | 37598 | 526 | DNA primase/helicase, phage-associated |
| CDS_57 | 37699 | 37890 | 63 | hypothetical protein |
| CDS_58 | 37887 | 38126 | 79 | hypothetical protein |
| CDS_59 | 38123 | 38338 | 71 | hypothetical protein |
| CDS_60 | 38335 | 38553 | 72 | hypothetical protein |
| CDS_61 | 38546 | 38761 | 71 | hypothetical protein |
| CDS_62 | 38761 | 38985 | 74 | hypothetical protein |
| CDS_63 | 38969 | 39139 | 56 | hypothetical protein |
| CDS_64 | 39296 | 39691 | 131 | hypothetical protein |
| CDS_65 | 39781 | 39993 | 70 | hypothetical protein |
| CDS_66 | 39993 | 40256 | 87 | hypothetical protein |
| CDS_67 | 40253 | 40438 | 61 | hypothetical protein |
| CDS_68 | 40477 | 40842 | 121 | hypothetical protein |
| CDS_69 | 40847 | 41065 | 72 | hypothetical protein |
| CDS_70 | 41078 | 41290 | 70 | hypothetical protein |
| CDS_71 | 41304 | 41747 | 147 | hypothetical protein |

**Supplementary Table 3c:** Features of *Enterococcus faecalis* bacteriophage vB_EfaS_140. The CDS gene number, and gene position in the phage vB_EfaS_140 genome are shown, as are the predicted length and function of the encoded products.

| Gene | Start (bp) | Stop (bp) | Length (aa) | Predicted function |
| --- | --- | --- | --- | --- |
| CDS_1 | 126 | 533 | 135 | HNH endocuclease |
| CDS_2 | 546 | 1277 | 243 | Phage-associated homing endonuclease |
| CDS_3 | 1368 | 1769 | 133 | Phage terminase, small subunit |
| CDS_4 | 1913 | 2209 | 98 | hypothetical protein with peroxydase motif |
| CDS_5 | 2571 | 4277 | 568 | Phage terminase, large subunit |
| CDS_6 | 4294 | 5574 | 426 | Phage portal protein |
| CDS_7 | 5602 | 6780 | 392 | Phage head maturation protease |
| CDS_8 | 6770 | 7972 | 400 | Phage major capsid |
| CDS_9 | 8284 | 9156 | 290 | hypothetical protein with lpxB motif |
| CDS_10 | 9313 | 9717 | 134 | DNA-packaging protein |
| CDS_11 | 9714 | 10076 | 120 | phage head-tail adaptor protein |
| CDS_12 | 10069 | 10518 | 149 | Phage protein |
| CDS_13 | 10521 | 10898 | 125 | hypothetical protein |
| CDS_14 | 10924 | 11568 | 214 | Phage major tail protein |
| CDS_15 | 11692 | 12024 | 110 | hypothetical protein |
| CDS_16 | 11999 | 12250 | 83 | hypothetical protein |
| CDS_17 | 12278 | 16084 | 1268 | Tail length tape measure protein |
| CDS_18 | 16084 | 16878 | 264 | Phage tail protein |
| CDS_19 | 16875 | 19139 | 754 | Phage endopeptidase |
| CDS_20 | 19202 | 21130 | 642 | hypothetical protein |
| CDS_21 | 21149 | 22720 | 523 | hypothetical protein |
| CDS_22 | 22767 | 23165 | 132 | hypothetical protein |
| CDS_23 | 23143 | 23487 | 114 | hypothetical protein |
| CDS_24 | 23502 | 23867 | 121 | Phage holin |
| CDS_25 | 23948 | 25090 | 380 | Phage lysin, N-acetylmuramoyl-L-alanine amidase |
| CDS_26 | 25159 | 26136 | 325 | Phage site-specific recombinase |
| CDS_27 | 26180 | 26740 | 186 | hypothetical protein |
| CDS_28 | 26740 | 26901 | 53 | hypothetical protein |
| CDS_29 | 26985 | 27191 | 68 | hypothetical protein |
| CDS_30 | 27224 | 27433 | 69 | hypothetical protein |
| CDS_31 | 27637 | 27777 | 46 | hypothetical protein |
| CDS_32 | 27857 | 28063 | 68 | hypothetical protein |
| CDS_33 | 28056 | 28460 | 134 | hypothetical protein |
| CDS_34 | 28450 | 28662 | 70 | hypothetical protein |
| CDS_35 | 28738 | 28947 | 69 | hypothetical protein |
| CDS_36 | 29068 | 29271 | 67 | hypothetical protein |
| CDS_37 | 29401 | 30105 | 234 | hypothetical aggregation promoting factor protein |
| CDS_38 | 30259 | 30447 | 62 | hypothetical protein |
| CDS_39 | 30526 | 30675 | 49 | hypothetical protein |
| CDS_40 | 30827 | 32815 | 662 | DNA girase subunit A/Topoisomerase IV |
| CDS_41 | 32816 | 33025 | 69 | hypothetical protein |
| CDS_42 | 33108 | 35048 | 646 | DNA gyrase subunit B |
| CDS_43 | 35097 | 35303 | 68 | hypothetical protein |
| CDS_44 | 35317 | 35514 | 65 | hypothetical protein |
| CDS_45 | 35529 | 36164 | 211 | hypothetical protein |
| CDS_46 | 36244 | 36591 | 115 | hypothetical protein |
| CDS_47 | 36693 | 36842 | 49 | hypothetical protein |
| CDS_48 | 36922 | 37056 | 44 | hypothetical protein |
| CDS_49 | 37171 | 37362 | 63 | hypothetical protein |
| CDS_50 | 37473 | 38021 | 182 | hypothetical protein |
| CDS_51 | 38102 | 38431 | 109 | hypothetical protein |
| CDS_52 | 38412 | 38987 | 191 | hypothetical protein |
| CDS_53 | 38987 | 39613 | 208 | Guanylate kinase |
| CDS_54 | 39610 | 39903 | 97 | hypothetical protein |
| CDS_55 | 39903 | 40604 | 233 | Deoxyuridine 5'-triphosphate nucleotidohydrolase |
| CDS_56 | 40604 | 40825 | 73 | hypothetical protein |
| CDS_57 | 40889 | 41167 | 92 | hypothetical protein |
| CDS_58 | 41185 | 41472 | 95 | hypothetical protein |
| CDS_59 | 41474 | 42019 | 181 | hypothetical protein |
| CDS_60 | 42009 | 42317 | 102 | hypothetical protein |
| CDS_61 | 42625 | 42894 | 89 | hypothetical protein |
| CDS_62 | 42887 | 43162 | 91 | hypothetical protein |
| CDS_63 | 43232 | 47086 | 1284 | DNA polymerase III alpha subunit |
| CDS_64 | 47169 | 47357 | 62 | hypothetical protein |
| CDS_65 | 47458 | 47673 | 71 | hypothetical protein |
| CDS_66 | 47684 | 47926 | 80 | hypothetical protein |
| CDS_67 | 47928 | 48536 | 202 | Thymidine kinase |
| CDS_68 | 48638 | 48829 | 63 | hypothetical protein |
| CDS_69 | 48822 | 49067 | 81 | hypothetical protein |
| CDS_70 | 49069 | 49206 | 45 | hypothetical protein |
| CDS_71 | 49226 | 49903 | 225 | Nucleotide sugar synthetase-like protein |
| CDS_72 | 50029 | 50553 | 174 | Ribonuclease HI |
| CDS_73 | 50625 | 51683 | 352 | beta glucosyl transferase |
| CDS_74 | 51693 | 52109 | 138 | Deoxyuridine 5'-triphosphate nucleotidohydrolase |
| CDS_75 | 52138 | 52983 | 281 | Thymidylate synthase |
| CDS_76 | 52977 | 53492 | 171 | hypothetical protein |
| CDS_77 | 53564 | 53764 | 66 | hypothetical protein |
| CDS_78 | 53766 | 53912 | 48 | hypothetical protein |
| CDS_79 | 53936 | 54130 | 64 | hypothetical protein |
| CDS_80 | 54206 | 54334 | 42 | hypothetical protein |
| CDS_81 | 54427 | 54897 | 156 | hypothetical protein |
| CDS_82 | 54890 | 55276 | 128 | hypothetical protein |
| CDS_83 | 55290 | 55898 | 202 | hypothetical protein |
| CDS_84 | 55995 | 57821 | 608 | Single-stranded-DNA-specific exonuclease RecJ |
| CDS_85 | 57832 | 58881 | 349 | DNA primase |
| CDS_86 | 58893 | 60473 | 526 | Replicative DNA helicase, DnaB |
| CDS_87 | 60470 | 60973 | 167 | hypothetical protein |
| CDS_88 | 61007 | 61381 | 124 | hypothetical protein |
| CDS_89 | 61396 | 61608 | 70 | hypothetical protein |
| CDS_90 | 61702 | 61929 | 75 | hypothetical protein |
| CDS_91 | 61942 | 62184 | 80 | hypothetical protein |
| CDS_92 | 62196 | 62420 | 74 | hypothetical protein |
| CDS_93 | 62460 | 63200 | 246 | hypothetical protein |
| CDS_94 | 63190 | 63399 | 69 | hypothetical protein |
| CDS_95 | 63396 | 63650 | 84 | hypothetical protein |
| CDS_96 | 63652 | 64314 | 220 | hypothetical protein |
| CDS_97 | 64425 | 65495 | 356 | ATP/GTP binding protein |
| CDS_98 | 65566 | 65991 | 141 | hypothetical protein |
| CDS_99 | 65991 | 66473 | 160 | hypothetical protein |
| CDS_100 | 66559 | 67527 | 322 | hypothetical protein |
| CDS_101 | 67627 | 67794 | 55 | hypothetical protein |
| CDS_102 | 67778 | 68422 | 214 | hypothetical protein |
| CDS_103 | 68415 | 69269 | 284 | hypothetical protein |
| CDS_104 | 69329 | 70366 | 345 | hypothetical protein |
| CDS_105 | 70457 | 70705 | 82 | hypothetical protein |
| CDS_106 | 70709 | 70987 | 92 | hypothetical protein |
| CDS_107 | 71059 | 71814 | 251 | NrdR-regulated deoxyribonucleotide transporter |
| CDS_108 | 71836 | 72672 | 278 | Deoxyadenosine/Deoxyguanosine kinase |
| CDS_109 | 72763 | 73395 | 210 | Purine trans deoxyribosylase |
| CDS_110 | 73397 | 73585 | 62 | hypothetical protein |
| CDS_111 | 73582 | 74130 | 182 | hypothetical protein |
| CDS_112 | 74227 | 74637 | 136 | hypothetical protein |
| CDS_113 | 74621 | 74833 | 70 | hypothetical protein |
| CDS_114 | 74852 | 75055 | 67 | hypothetical protein |
| CDS_115 | 75145 | 75360 | 71 | hypothetical protein |
| CDS_116 | 75545 | 75736 | 63 | hypothetical protein |
| CDS_117 | 75751 | 77037 | 428 | DNA ligase |
| CDS_118 | 77069 | 77302 | 77 | hypothetical protein |
| CDS_119 | 77305 | 77559 | 84 | hypothetical protein |
| CDS_120 | 77559 | 77996 | 145 | hypothetical protein |
| CDS_121 | 77993 | 78304 | 103 | hypothetical protein |
| CDS_122 | 78415 | 78537 | 40 | hypothetical protein |
| CDS_123 | 78745 | 79239 | 164 | hypothetical protein |
| CDS_124 | 79250 | 81214 | 654 | RecD-like DNA helicase |
| CDS_125 | 81302 | 82900 | 532 | hypothetical protein |
| CDS_126 | 82904 | 83110 | 68 | hypothetical protein |
| CDS_127 | 84291 | 85154 | 287 | hypothetical protein |
| CDS_128 | 85144 | 85299 | 51 | hypothetical protein |

**Supplementary Table 3d:** Features of *Enterococcus faecalis* bacteriophage vB_EfaS_159. The CDS gene number, and gene position in the phage vB_EfaS_159 genome are shown, as are the predicted length and function of the encoded products.

| Gene | Start (bp) | Stop (bp) | Length (aa) | Predicted function |
| --- | --- | --- | --- | --- |
| CDS_1 | 288 | 500 | 70 | hypothetical protein |
| CDS_2 | 514 | 714 | 66 | hypothetical protein |
| CDS_3 | 714 | 962 | 82 | hypothetical protein |
| CDS_4 | 962 | 1357 | 131 | Putative homing endonuclease |
| CDS_5 | 1697 | 1870 | 57 | hypothetical protein |
| CDS_6 | 1885 | 2361 | 158 | Phage terminase, small subunit |
| CDS_7 | 2962 | 4689 | 575 | Phage terminase, large subunit |
| CDS_8 | 4778 | 5044 | 88 | hypothetical protein |
| CDS_9 | 5119 | 5283 | 54 | hypothetical protein |
| CDS_10 | 5288 | 6466 | 392 | Phage portal protein |
| CDS_11 | 6441 | 6995 | 184 | Phage head maturation protease |
| CDS_12 | 7072 | 8292 | 406 | Major capsid protein |
| CDS_13 | 8418 | 8621 | 67 | hypothetical protein |
| CDS_14 | 8663 | 8956 | 97 | Head-tail connector protein |
| CDS_15 | 8931 | 9263 | 110 | Head-tail connector protein |
| CDS_16 | 9260 | 9667 | 135 | Putative head-tail joining protein |
| CDS_17 | 9664 | 10029 | 121 | Head-tail joining protein |
| CDS_18 | 10108 | 10674 | 188 | Major tail protein |
| CDS_19 | 10849 | 11181 | 110 | Putative tail tape measure chaperone protein |
| CDS_20 | 11247 | 11420 | 57 | Putative tail tape measure chaperone protein |
| CDS_21 | 11420 | 15286 | 1288 | Phage tail length tape-measure protein |
| CDS_22 | 15305 | 17380 | 691 | Minor tail protein |
| CDS_23 | 17425 | 19653 | 742 | Phage tail assembly |
| CDS_24 | 19834 | 20079 | 81 | Phage tail fiber protein |
| CDS_25 | 20094 | 20327 | 77 | Phage holin |
| CDS_26 | 20330 | 21427 | 365 | Phage lysin, N-acetylmuramoyl-L-alanine amidase |
| CDS_27 | 21517 | 21744 | 75 | Glutaredoxin-like protein |
| CDS_28 | 21745 | 22581 | 278 | hypothetical protein |
| CDS_29 | 22644 | 24935 | 763 | DNA polymerase B region |
| CDS_30 | 24971 | 25699 | 242 | Putative DNA methylase |
| CDS_31 | 25740 | 25916 | 58 | hypothetical protein |
| CDS_32 | 25968 | 26192 | 74 | hypothetical protein |
| CDS_33 | 26263 | 26970 | 235 | hypothetical protein |
| CDS_34 | 27049 | 27300 | 83 | hypothetical protein |
| CDS_35 | 27301 | 27591 | 96 | hypothetical protein |
| CDS_36 | 27588 | 28409 | 273 | hypothetical protein |
| CDS_37 | 28399 | 28587 | 62 | hypothetical protein |
| CDS_38 | 28563 | 29339 | 258 | hypothetical protein |
| CDS_39 | 29350 | 29829 | 159 | hypothetical protein |
| CDS_40 | 29826 | 30032 | 68 | hypothetical protein |
| CDS_41 | 30034 | 30540 | 168 | hypothetical protein |
| CDS_42 | 30694 | 30900 | 68 | hypothetical protein |
| CDS_43 | 30893 | 31639 | 248 | Adenine-specific methyltransferase |
| CDS_44 | 31632 | 32375 | 247 | Putative bifunctional DNA primase/polymerase |
| CDS_45 | 32387 | 32575 | 62 | hypothetical protein |
| CDS_46 | 32634 | 32810 | 58 | hypothetical protein |
| CDS_47 | 32807 | 34102 | 431 | hypothetical protein |
| CDS_48 | 34095 | 34484 | 129 | hypothetical protein |
| CDS_49 | 34468 | 34701 | 77 | hypothetical protein |
| CDS_50 | 34704 | 34913 | 69 | hypothetical protein |
| CDS_51 | 34915 | 35076 | 53 | hypothetical protein |
| CDS_52 | 35078 | 35350 | 90 | hypothetical protein |
| CDS_53 | 35399 | 35860 | 153 | hypothetical protein |
| CDS_54 | 35935 | 36105 | 56 | hypothetical protein |
| CDS_55 | 36179 | 36367 | 62 | hypothetical protein |
| CDS_56 | 36456 | 38036 | 526 | DNA primase/helicase |
| CDS_57 | 38137 | 38328 | 63 | hypothetical protein |
| CDS_58 | 38325 | 38564 | 79 | hypothetical protein |
| CDS_59 | 38561 | 38776 | 71 | hypothetical protein |
| CDS_60 | 38773 | 38991 | 72 | hypothetical protein |
| CDS_61 | 38988 | 39200 | 70 | hypothetical protein |
| CDS_62 | 39200 | 39424 | 74 | hypothetical protein |
| CDS_63 | 39424 | 39582 | 52 | hypothetical protein |
| CDS_64 | 39734 | 40129 | 131 | hypothetical protein |
| CDS_65 | 40143 | 40349 | 68 | hypothetical protein |
| CDS_66 | 40439 | 40651 | 70 | hypothetical protein |
| CDS_67 | 40651 | 40917 | 88 | hypothetical protein |
| CDS_68 | 40914 | 41099 | 61 | hypothetical protein |
| CDS_69 | 41138 | 41503 | 121 | hypothetical protein |
